# Mechanisms, injury patterns, and kinematic features of hamstring strain injuries in football (soccer): a systematic review and meta-analysis of video-analysis studies

**DOI:** 10.3389/fpubh.2026.1846524

**Published:** 2026-05-21

**Authors:** Jingkang Xia, Junhao He, Jiayi Yao, Yongliang Zhu, Lihong Mao, Xueqiang Zhu

**Affiliations:** 1 School of Competitive Sport, Shandong Sport University, Rizhao, China; 2 School of Physical Education and Training, Capital University of Physical Education and Sports, Beijing, China; 3 School of Physical Education, China University of Mining and Technology, Xuzhou, China

**Keywords:** biomechanics, football (soccer), hamstring strain injury, injury mechanism, meta-analysis, video analysis

## Abstract

**Background and objective:**

Match video analysis enables retrospective examination of the movement tasks, match situations, and body postures immediately before and after hamstring strain injury (HSI) in football, thereby providing information on the injury process that is difficult to obtain directly from conventional clinical or imaging data. This study aimed to systematically evaluate video-based studies of football matches and to compare the relative reporting frequencies of several prespecified key epidemiological contrasts. In addition, single-arm proportion pooling was used to provide descriptive background information, and exploratory analyses were conducted on kinematic postures and sex differences.

**Methods:**

Seven databases were systematically searched from inception to February 12, 2026. Studies of 11-a-side football were included if they used video analysis to assess the inciting moment of real-match HSI and reported injury mechanisms, situational patterns, or kinematic characteristics. The primary analyses focused on five prespecified pairwise comparisons. Study-specific 2 × 2 contingency tables were constructed, and odds ratios (ORs) with 95% confidence intervals (CIs) were pooled using random-effects models. The primary OR analyses were restricted to male samples. Single-arm proportions were pooled using a generalized linear mixed model. Analyses related to kinematics and sex were all treated as exploratory.

**Results:**

A total of 7 studies involving 342 HSI cases were included. The main comparisons showed that non-contact was reported more frequently than indirect contact (OR = 2.51, 95% CI 1.31, 4.82), and sprint-type was reported more frequently than mixed-type (OR = 2.01, 95% CI 1.04, 3.87). In contrast, sprint vs. stretch, offensive vs. defensive, and first half vs. second half showed no clear differences. The descriptive pooled results suggested relatively high pooled proportions for sprint-type, running-related action, and non-contact. Exploratory analyses showed that samples associated with different mechanisms were generally similar in knee-joint posture, whereas differences may exist in hip-joint and trunk alignment; cross-study exploratory comparisons by sex did not identify any results that remained consistently significant after multiple-comparison correction.

**Conclusion:**

Existing video-analysis evidence from football matches shows that HSI occurs more commonly in situations without external contact, and that sprint-type HSI is more frequent than mixed-type HSI; however, the relative differences between offensive and defensive phases and between the first and second halves remain unclear. These findings may provide a basis for hypotheses in football HSI prevention research, suggesting that future studies may consider examining non-contact injury scenarios in the context of high-speed running, eccentric load tolerance, and neuromuscular control during the single-leg support phase; however, the existing video-analysis evidence itself cannot directly demonstrate the preventive effects of specific training strategies. The above conclusions are limited by factors such as the small number of included studies, inconsistent definitions of injury mechanisms, and insufficient standardization of video analysis, and future research should further accumulate cross-sex samples and harmonize reporting standards.

**Systematic review registration:**

This systematic review and meta-analysis has been registered in PROSPERO (www.crd.york.ac.uk/prospero), identifier CRD420261335634.

## 1 Introduction

Hamstring strain injury (HSI) is one of the most common time-loss muscle injuries in competitive sport and is associated with a high risk of recurrence (1, 2). In professional football, this type of injury also affects players’ training continuity and match availability and may impair performance in the early period after return to play (3, 4). In long-term longitudinal surveillance of professional men’s football, hamstring injuries accounted for approximately 19% of all reported injuries, with their proportional share increasing from 12% in the 2001/02 season to 24% in the 2021/22 season; over the same period, the proportion of absence days attributable to these injuries also increased from 10 to 20% (3). This suggests that HSI not only occurs frequently, but that its relative contribution to the overall injury burden in professional football continues to increase.

To more comprehensively understand the injury process of HSI, reliance solely on imaging assessment, clinical diagnosis, or players’ self-reports often makes it difficult to reconstruct the actual movement context at the moment of injury (5, 6). Match video analysis can provide complementary information on the movement task, body alignment, and contact context before and after injury occurrence, but its results remain influenced by video quality, interpretation criteria, and classification frameworks (5–7). Previous football video studies have suggested that HSI is commonly associated with sprinting, high-speed running, kicking, and actions involving excessive lengthening; however, most existing studies used observational designs, and the definitions of mechanisms, contextual classifications, and sex distribution of samples are not fully consistent (6–8). Therefore, a focused synthesis is needed to compare prespecified epidemiological contrasts across football HSI video studies.

Accordingly, this study aimed to systematically evaluate studies using match video analysis in football and to address the following questions through meta-analysis: (1) Are there differences in the relative reporting frequencies across contact modes (non-contact vs. indirect contact) and mechanism types (sprint-type vs. stretch-type vs. mixed-type)? (2) Is the distribution of HSI uneven between offensive and defensive phases and between the first and second halves? (3) Do samples associated with different mechanisms differ in kinematic posture at the moment of injury? In addition, this study used single-arm proportion pooling to provide descriptive background information on event proportions across different dimensions and conducted exploratory comparisons of epidemiological and kinematic differences between male and female players.

## 2 Methods

The study protocol was registered in PROSPERO after completion of the preliminary database search but before formal title/abstract screening, full-text eligibility assessment, data extraction, risk-of-bias assessment, and statistical analysis (registration number: CRD420261335634) (9). Therefore, the registration was not completed before the preliminary search, but it preceded study selection and all data-dependent review procedures. This study was reported in accordance with the PRISMA 2020 statement, with reference to the MOOSE statement to supplement relevant reporting items for systematic reviews and meta-analyses of observational studies (10, 11).

### 2.1 Search strategy and eligibility criteria

The search period covered each database from inception to February 12, 2026. No restriction was applied to publication year or publication period; therefore, records from all available years in each database were eligible up to the final search date. Seven electronic databases were systematically searched: PubMed, SPORTDiscus, Web of Science, Scopus, Embase, the Cochrane Library, and CINAHL. The search strategy was constructed around three core concepts: “hamstring injury,” “video analysis,” and “football/soccer,” and was adapted to the search interface of each database. The full search strategies for all databases are presented in Supplementary Text S1.

**Figure 1:**
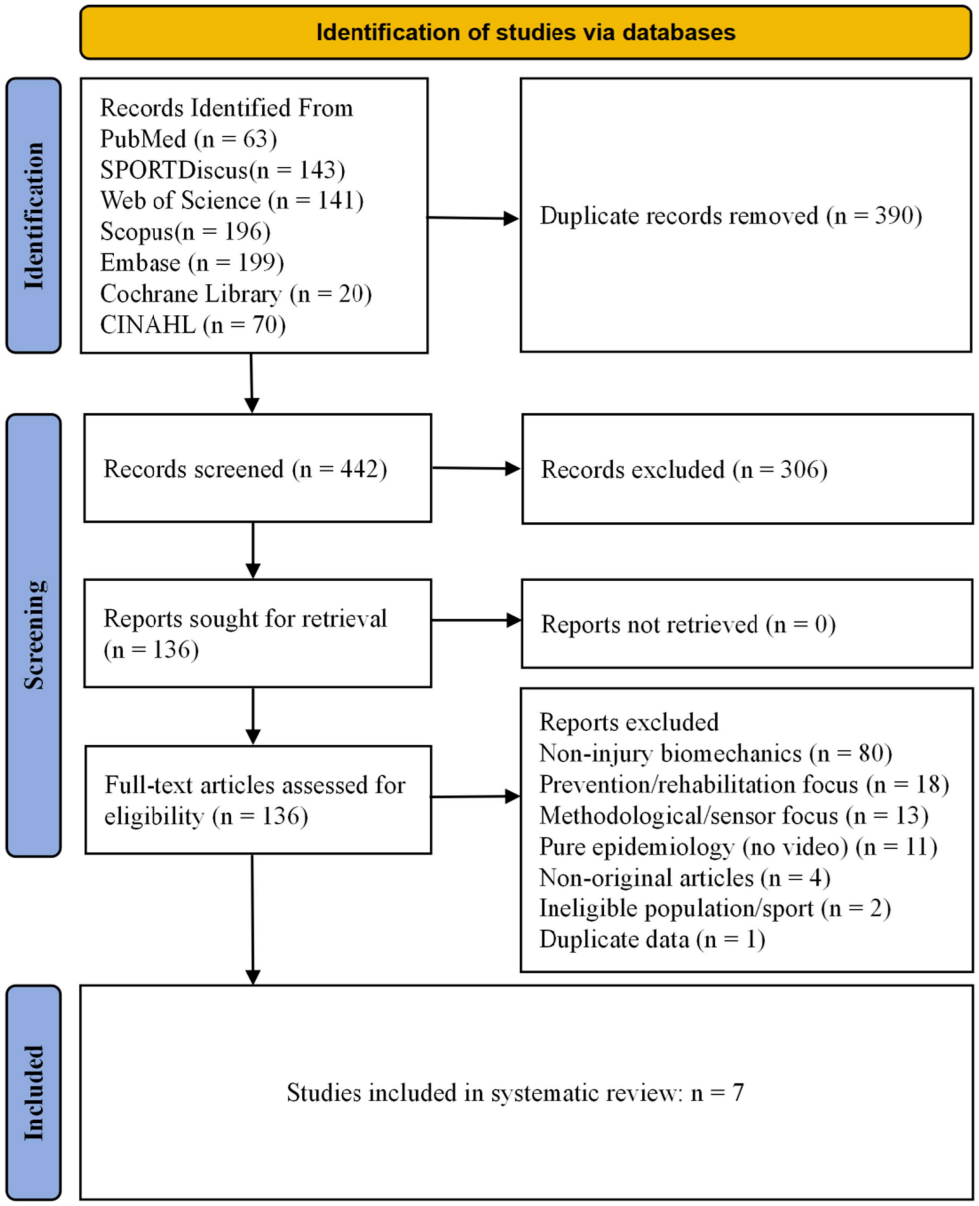
PRISMA 2020 flow diagram of study identification, screening, eligibility assessment, and inclusion.

Because this review synthesized observational video-analysis studies and evaluated injury events, mechanisms, and contextual phenomena rather than assigned interventions, the eligibility criteria were structured according to an adapted PECOS framework, including Population, Exposure/Phenomenon of interest, Comparator/grouping, Outcomes, and Study design, as detailed in Table 1. The literature screening process is shown in Figure 1.

**Table 1:** Eligibility criteria according to an adapted PECOS framework.

PECOS item	Inclusion criteria	Exclusion criteria or handling rules
Population	11-a-side football players, regardless of sex, age category, or competitive level; the study population was required to include HSI events	Non-football sports; non-11-a-side football; studies in which football samples or HSI samples could not be separately distinguished
Exposure/phenomenon of interest	HSI injury events occurring during actual matches, with video analysis used to assess the moment of injury or the context before and after injury occurrence	Training or laboratory-simulated tasks; studies based only on questionnaires, interviews, medical records, or imaging data, without video analysis of actual matches
Comparator/grouping	No external control group was required; categorical comparisons according to mechanism, contact type, movement task, match context, time period, playing position, or sex were allowed	Studies with no extractable category counts or category descriptions and not usable for descriptive pooling or comparative analysis
Outcomes	At least one of the following dimensions was reported: injury mechanism, contact situation, movement task, match context, temporal distribution, playing position, or kinematic posture	Studies that did not report HSI injury mechanisms, contextual patterns, or kinematic features
Study design	Original observational video-analysis studies, including case series, cross-sectional descriptive studies, or retrospective video-analysis studies	Reviews, editorials, commentaries, methodological papers, conference abstracts, and non-original studies; for duplicate or partially overlapping datasets, only the most complete source of information was retained, and overlapping data were not counted repeatedly in the analysis

### 2.2 Data extraction

Literature screening, data extraction, data organization, and quality assessment were independently performed by two reviewers (YLZ and JKX). Disagreements during screening, extraction, or assessment were first resolved through discussion; when consensus could not be reached, a third reviewer (LHM) made the final decision. The extracted data included basic study characteristics, injury mechanisms (sprint-type, stretch-type, and mixed-type), action categories (e.g., running, kicking, change of direction, and reaching/stretching for the ball), tactical situations, contact characteristics, temporal distribution, positional distribution, and kinematic variables (high-speed status as well as the sagittal-plane postures of the knee, hip, and trunk at the moment of injury).

For differences across studies in joint-angle binning, particularly knee-joint angle binning, all data were first entered item by item according to the original reports, without forced harmonization at the data-extraction stage; recoding and sensitivity handling were performed only when cross-study comparisons were subsequently required, according to prespecified rules. Mechanism type was preferentially extracted according to the original study reports. Sprint-type referred to injuries occurring during sprinting, acceleration, or high-speed running-related tasks, primarily characterized by active high-speed stretching or eccentric loading within the running cycle; stretch-type referred to injuries occurring during kicking, slide tackling, reaching for the ball with the leg, lunging, or other excessive lengthening situations leading to increased hip flexion and/or knee extension; mixed-type referred to cases in which the original study explicitly used a mixed-mechanism classification, or in which the same event had both sprint/running and stretch/overstretch characteristics and could not be reliably classified as either sprint-type or stretch-type alone. For events in which the original study did not use mixed-type but the description suggested unclear boundaries, this study did not subjectively create a new mechanism category; instead, the original study classification was retained, and this was explained in sensitivity or descriptive interpretation.

### 2.3 Risk-of-bias and methodological quality assessment

The included studies were evaluated across three dimensions: risk of bias, reporting completeness, and methodological quality of video analysis. Risk of bias was assessed using the JBI Critical Appraisal Checklist for Case Series (12); reporting completeness was evaluated using the core reporting item set pre-specified in this study based on STROBE-SIIS (13, 14); and the methodological quality of video analysis was assessed using the QA-SIVAS scale (7). The three assessments were conducted and reported independently, without generating a combined score across tools. Complete item-level ratings are presented in Supplementary Tables S1–S3.

Risk of bias was assessed using the JBI Critical Appraisal Checklist for Case Series (12). This tool was selected because all included studies used existing HSI cases or injury events as the sampling framework and primarily described their mechanisms, contexts, and postural features as interpreted through video analysis, rather than estimating incidence risk based on follow-up of exposed and non-exposed groups. The JBI case series checklist contains 10 items covering aspects such as criteria for case inclusion, methods of injury identification, consecutive and complete inclusion of cases, reporting of sample characteristics, reporting of clinical/contextual information, and appropriateness of statistical analysis. Each item was scored as 1 point for “yes” and 0 points for “no/unclear”; items rated as “not applicable” were excluded from the denominator, and the percentage score was calculated accordingly. According to the operational rules prespecified in this study, a score rate of ≥80% was defined as low risk of bias, 60–79% as moderate risk of bias, and <60% as high risk of bias.

Reporting completeness was assessed using eight core reporting items pre-specified in this study based on STROBE-SIIS (13, 14). Each item was scored as 1 for “completely reported,” 0.5 for “partially reported,” and 0 for “not reported”; items rated as not applicable were excluded from the denominator, and the item completion rate was calculated accordingly. According to the prespecified rules of this study, a completion rate of ≥80% was defined as complete reporting, 60–79% as moderate completeness, and <60% as insufficient reporting.

The methodological quality of video analysis was assessed using the QA-SIVAS scale (7). Each item was scored as 1 for “yes” and 0 for “no/not stated,” and the percentage score was calculated as the total score relative to the maximum possible score. With reference to the interpretive ranges recommended in the original QA-SIVAS study (7), 81–100% was defined as high quality, 71–80% as good quality, 60–70% as moderate quality, and <60% as low quality.

### 2.4 Statistical analysis

Before statistical analysis, the extracted information was organized into three structured datasets for study-level epidemiological summaries, position–mechanism mapping, and biomechanical variable analyses, respectively. “Not reported” (NR), blank cells, and other unreported values in the raw data were uniformly coded as missing values, and variable names, category labels, and the format of count variables were standardized. Missing information was handled according to the analytical dimension: when a study did not report a specific dimension, it was excluded only from the analysis of that dimension but remained eligible for analyses of other dimensions. No statistical imputation was performed for missing counts, except for prespecified scenario-based recoding when knee joint angle binning was inconsistent. In cases of numerator–denominator inconsistency, programmatic checks were first conducted, followed by harmonization based on dimension-specific denominators.

Statistical analyses included main comparative analyses and descriptive pooled analyses. This study prespecified five epidemiological pairwise comparisons as the main OR comparisons, based on their mechanistic interpretive value, practical relevance, and cross-study extractability. These comparisons corresponded to core dimensions that are commonly reported in football HSI video studies and have clear interpretive meaning, including mechanism type, contact type, tactical phase, and match period, and could be used to construct 2 × 2 tables based on mutually exclusive category counts across multiple studies. Accordingly, the main comparative analyses included sprint-type vs. stretch-type, sprint-type vs. mixed-type, non-contact vs. indirect contact, offensive vs. defensive, and first half vs. second half. Other variables were handled only as descriptive, supplementary, or exploratory analyses because of inconsistent reporting formats, non-uniform category boundaries, or sparse data. For each comparison, the logarithm of the odds ratio (log[OR]) and its standard error were calculated within each study based on the counts of two mutually exclusive categories within the same analytical dimension, and pooled using a random-effects model, with between-study variance estimated by the DerSimonian–Laird method (15). If any cell contained 0, the Haldane–Anscombe 0.5 continuity correction was applied only to the 2 × 2 contingency table of that study (i.e., 0.5 was added to each of the four cells) (16). The direction of the OR was defined as the former category relative to the latter category. The main OR analyses were restricted to studies of male players; female football samples were presented separately.

Descriptive pooled analyses were used for single-arm proportion pooling. All variables were pooled using a generalized linear mixed model (GLMM) based on a logit link, with a random-effects model fitted in R using the rma.glmm function of the metafor package (measure = “PLO”); the random-effects model was estimated by maximum likelihood (17–19). When GLMM fitting failed, a random-effects single-arm proportion model based on logit transformation was used as a supplementary implementation. For each variable, the pooled proportion, 95% CI, *I*^2^, and *τ*^2^ were reported; for variables with *I*^2^ > 75%, a 95% prediction interval was additionally reported, and leave-one-out analysis was performed (20). To address reporting differences across studies within each analytical dimension, dimension-specific denominators were used consistently; when multiple coding existed within the same dimension, the denominator was defined as the total number of events actually reported for that dimension, rather than being uniformly traced back to the overall number of HSIs. Dynamic-denominator diagnostics are shown in Supplementary Figure S3.

The kinematic-posture contrast between sprint- and stretch-related samples was presented only descriptively. Sex-stratified results were prespecified as exploratory analyses, mainly because the number of female samples and analyzable videos was limited, and because male and female samples were imbalanced in terms of the number of studies, competition sources, season coverage, video availability, and injury definitions. Therefore, sex-stratified results were used only to describe potential trends and generate hypotheses, rather than for confirmatory inference or causal interpretation. In the epidemiological dimension, exact binomial estimates were used for the single female study and descriptively compared with the 95% CI and the 95% prediction interval from the male main analysis; in the kinematic dimension, Fisher’s exact test was used to calculate *p* values, and absolute risk differences (ARDs) and their 95% CIs were reported, with supplementary reporting of ORs and their 95% CIs based on the Woolf logit approximation. If 0 cells were present, calculations were performed after applying a 0.5 continuity correction (16, 21). The Benjamini–Hochberg method was used to control the false discovery rate for the 5 prespecified variables (22). To address inconsistency in knee-joint angle binning, the original study bins were retained, and sensitivity analyses were conducted under 3 recoding scenarios: Min, Uniform, and Max. Because the number of studies included in each OR comparison was small, funnel plots were presented only as supplementary visualizations and were not used for formal statistical inference regarding publication bias (23).

## 3 Results

### 3.1 Search results

A total of 832 records were identified, and 442 records entered title and abstract screening after deduplication. After 306 records were excluded, 136 full-text articles were assessed for eligibility, and all full texts were successfully retrieved. Ultimately, 129 full-text articles were excluded for the following reasons: non-injury biomechanical studies (*n* = 80), prevention- or rehabilitation-oriented studies (*n* = 18), methodological or sensor studies (*n* = 13), non-video epidemiological studies (*n* = 11), non-original studies (*n* = 4), studies not meeting the sport or population criteria (*n* = 2), and duplicate data (*n* = 1). Finally, seven studies were included. The literature search and screening process is shown in Figure 1.

### 3.2 Characteristics of the included studies

The included studies were published between 2022 (24) and 2025 (25, 26), and the study periods covered 2013 (27) to 2024 (25). A total of 342 HSIs were included, with sample sizes ranging from 14 (28) to 78 (26) per study. The study populations all comprised professional or professional/elite-level football players, including six studies with male samples (24, 26–30) and one study with a female sample (25). Data sources involved professional leagues or competition systems in the United States (25), Italy (29, 30), Spain (25, 26, 28), England (25), France (25), Germany (24, 25), Finland (28), and Qatar (27). All studies were based on match video and used two-dimensional video analysis, primarily to identify injury mechanisms, match situations, and related kinematic characteristics at the time of HSI occurrence. The basic characteristics of the included studies are presented in Table 2, and details of the video analysis procedures are provided in Supplementary Table S4.

**Table 2:** Characteristics of the included studies.

Study	Sex	Country/region	Competition/league	Study period	Level	HSI, *n*	Videos retained/excluded
Pellegrini, 2025 (25)	F	USA/ITA/ESP/ENG/FRA/GER	Domestic and Int. (UCL, Cups)	2017–2024	Prof/Elite	57	57/52
Jokela, 2023 (28)	M	FIN/ESP	NR (Prof leagues)	2017–2022	Prof	14	14/NR
Vermeulen, 2024 (27)	M	QAT	QSL	2013–2020	Prof	63	63/68
Della Villa, 2023 (29)	M	ITA	Serie A and Cups	2018–2021	Prof	61	103/18
Gandarias-Madariaga, 2025 (26)	M	ESP	LaLiga	2017–2019	Prof	78	78/47
Gronwald, 2022 (24)	M	GER	Bundesliga (BL1/BL2)	2014–2019	Prof	52	52/56
Aiello, 2023 (30)	M	ITA	Serie A	3 seasons	Prof	17	17/NR

M = male; F = female; Prof = professional; Elite = elite; HSI = hamstring strain injury; NR = not reported; UCL = UEFA Champions League; QSL = Qatar Stars League; BL1/BL2 = Bundesliga 1/2. “Videos retained/excluded” indicates the numbers of videos retained for analysis and excluded after video review, respectively. Della Villa, 2023 initially reported 103 lower-limb injury videos, of which 61 were confirmed HSI cases and were included in this review. Aiello, 2023 reported data from three consecutive seasons without specifying the calendar years. Country codes, where applicable, follow FIFA-standard abbreviations. For multi-country studies, country codes indicate the countries or competition systems contributing analyzable videos.

### 3.3 Quality assessment

The results of the quality assessment are shown in Table 3. According to the prespecified operational rules of this study, the JBI assessment indicated that, among the seven studies, three were at moderate risk of bias and four were at high risk of bias; the assessment based on the core STROBE-SIIS items showed that three studies had complete reporting and four had moderate reporting completeness; the QA-SIVAS assessment showed that five studies were of high quality, one was of good quality, and one was of moderate quality. In terms of reporting completeness, all studies reached at least a moderate level. In terms of methodological quality of video analysis, six studies reached at least good quality. In terms of risk of bias, all studies were at moderate-to-high risk. Item-level ratings for each tool are provided in Supplementary Tables S1–S3 (7, 12–14).

**Table 3:** Summary of risk of bias, reporting completeness, and video-analysis methodological quality of the included studies.

Study	JBI	STROBE-SIIS	QA-SIVAS
Pellegrini, 2025 (26)	5/10; moderate	6.5/8; 81.2%; complete	14/18; 77.8%; good
Jokela, 2023 (28)	5/10; moderate	6/8; 75.0%; moderate	15/18; 83.3%; high
Vermeulen, 2024 (27)	4/10; high	5.5/8; 68.8%; moderate	15/18; 83.3%; high
Della Villa, 2023 (29)	5/10; moderate	5.5/8; 68.8%; moderate	15/18; 83.3%; high
Gandarias-Madariaga et al., 2025 (26)	4/10; high	6/8; 75.0%; moderate	12/18; 66.7%; moderate
Gronwald et al., 2022 (24)	4/10; high	6.5/8; 81.2%; complete	16/18; 88.9%; high
Aiello et al., 2023 (30)	4/10; high	6.5/8; 81.2%; complete	15/18; 83.3%; high

JBI was used to assess risk of bias, STROBE-SIIS to assess reporting completeness, and QA-SIVAS to assess the methodological quality of video-analysis studies. In the JBI column, “moderate” and “high” refer to the risk-of-bias grade; in the STROBE-SIIS and QA-SIVAS columns, they refer to reporting completeness and video-analysis methodological quality, respectively. Values are presented as score/maximum and, where applicable, percentage plus grade. Item-level results for each tool are provided in Supplementary Tables S1–S3.

### 3.4 Results of the primary comparative analyses

**Figure 2:**
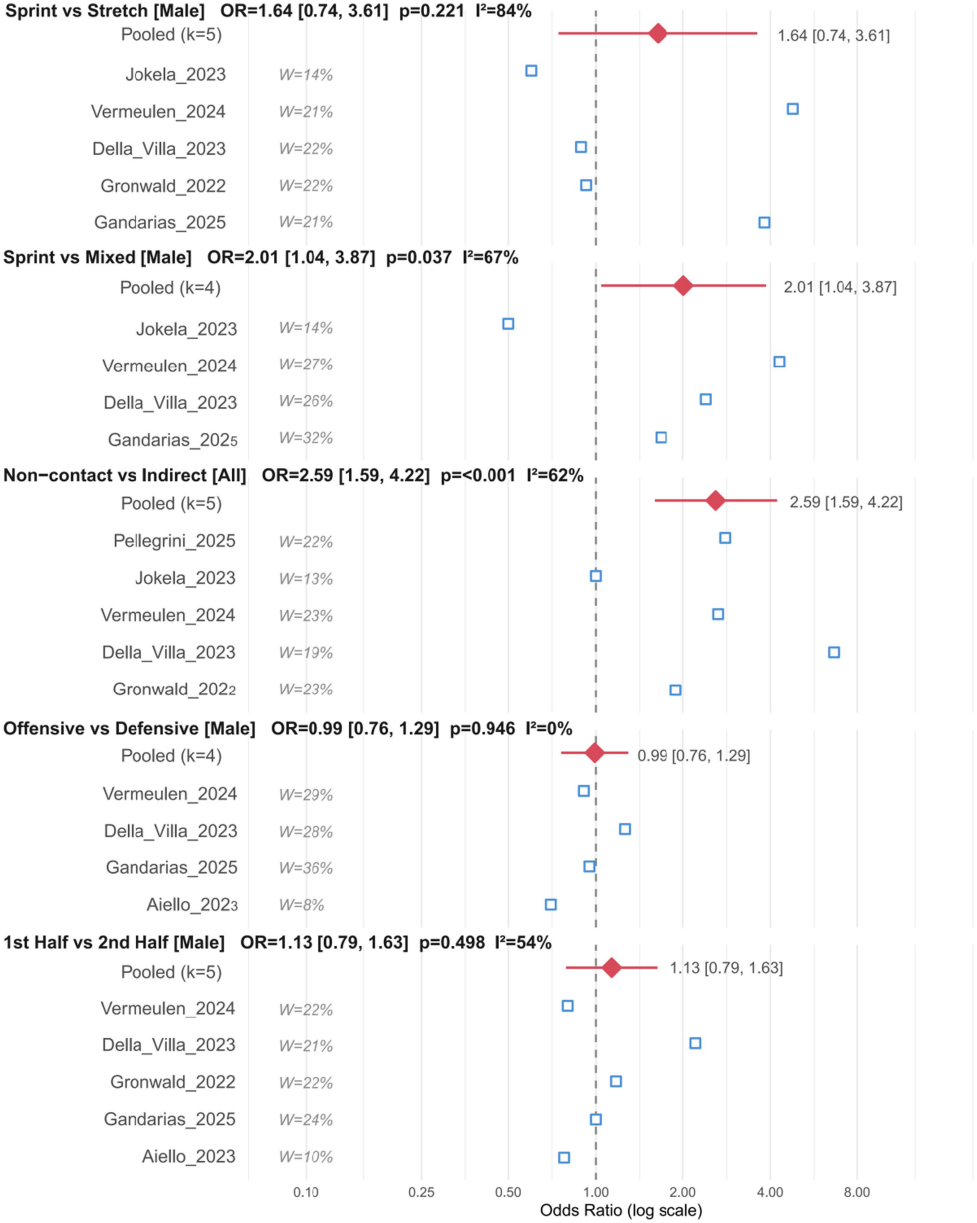
Forest plots for the five prespecified pairwise OR comparisons. Forest plots for the five prespecified pairwise odds-ratio (OR) comparisons: Sprint-type vs. stretch-type; sprint-type vs. mixed-type; non-contact vs. indirect contact; offensive vs. defensive; and first half vs. second half. Squares represent study-specific ORs, with square size proportional to study weight; diamonds represent pooled random-effects estimates; horizontal lines indicate 95% confidence intervals (CIs); and the vertical dashed line indicates the null value (OR = 1.0). Pooled estimates were calculated using the DerSimonian–Laird method. For each comparison, the number of included studies (*k*), pooled OR, 95% CI, *p* value, and *I*^2^ are reported. OR > 1 indicates that, within the same analytic domain and analyzable event set, the first category was reported more frequently than the second.

The following five prespecified OR comparisons constituted the main quantitative analyses of this study; all remaining single-group rates, kinematic postures, sex comparisons, and sensitivity analyses were used for descriptive supplementation or hypothesis generation and were not used as the basis for confirmatory inference. The results of the main OR comparative analyses are shown in Figure 2; forest plots for the other 6 supplementary OR comparisons are provided in Supplementary Figure S1, and individual forest plots for all 11 comparisons are provided in Supplementary Figure S2.

For comparisons related to contact type and mechanism, the male primary analysis showed a higher pooled OR for non-contact than for indirect contact (OR = 2.51, 95% CI 1.31, 4.82, *p* = 0.006, *I*^2^ = 71%) (31, 32). In the comparison of mechanism types, the pooled OR for sprint-type versus mixed-type was also higher (OR = 2.01, 95% CI 1.04, 3.87, *p* = 0.037, *I*^2^ = 67%) (31, 32). By contrast, no clear difference was observed for sprint vs. stretch (OR = 1.64, 95% CI 0.74, 3.61, *p* = 0.221), and heterogeneity was high (*I*^2^ = 84%), indicating inconsistent results across studies for this comparison (8, 20, 33).

For comparisons of match situations and temporal distribution, the OR for offensive vs. defensive was 0.99 (95% CI 0.76, 1.29, *p* = 0.946, *I*^2^ = 0%), indicating similar relative reporting frequencies between offensive and defensive phases, with no observed heterogeneity; the OR for first half vs. second half was 1.13 (95% CI 0.79, 1.63, *p* = 0.498, *I*^2^ = 54%), indicating no clear relative difference between the two halves (31–33). Overall, among the five prespecified comparisons, the primary analyses identified higher relative reporting frequencies for non-contact versus indirect contact and for sprint-type versus mixed-type, whereas no clear differences were observed for sprint-type versus stretch-type, offensive versus defensive, or first half versus second half (31–33).

### 3.5 Descriptive pooled results

To further present the distributional characteristics of event proportions across dimensions and to provide descriptive background for the OR comparisons above, the results of the single-arm proportion pooling are reported below (17, 20, 34). In the mechanism dimension, sprint-type had the highest pooled proportion at 48.8% (95% CI 36.4, 61.4%, *I*^2^ = 73.2%), followed by stretch-type at 29.4% (95% CI 16.7, 46.4%, *I*^2^ = 83.9%) and mixed-type at 23.8% (95% CI 15.8, 34.2%, *I*^2^ = 55.0%). The distribution of these three mechanism categories was consistent with the direction of the primary OR comparison showing a higher reporting frequency for sprint-type than for mixed-type. In the action and contact dimensions, the pooled proportion was 86.6% for running-related action (95% CI 47.0, 97.9%, *I*^2^ = 95.1%) and 89.4% for non-contact (95% CI 58.7, 98.0%, *I*^2^ = 94.9%); however, the *I*^2^ values for both variables exceeded 90%, indicating substantial between-study variation and limiting the interpretability and generalizability of the pooled estimates. Regarding temporal distribution, the pooled proportions for the first and second halves were 53.3 and 46.7%, respectively. When divided into six 15-min periods (0–15, 16–30, 31–45, 46–60, 61–75, and 76–90 min), the pooled proportions across periods ranged from 14.1 to 18.2%, broadly consistent with the first-half versus second-half OR comparison, suggesting no obvious concentration of HSI events within a specific match period. Overall, these proportion-based results were directionally consistent with the primary OR comparisons; however, given the high heterogeneity for some variables, particularly running-related action and non-contact, their interpretation should remain primarily descriptive (17, 20, 34).

### 3.6 Exploratory results

#### 3.6.1 Descriptive comparison of kinematic postures

In the descriptive comparison of kinematic postures, the samples from the two studies with extractable relevant data showed relatively consistent directions in knee-joint posture, with knee flexion <45° at the moment of injury predominating in both studies. By contrast, the distributions of hip-joint and trunk postures suggested that certain differences may exist, with representative sprint/mixed samples more commonly showing hip flexion, whereas the stretch-type sample was not fully consistent with them in the distributions of hip-joint and trunk postures (5, 6, 8). However, because the number of comparable studies was limited, one study sample did not consist of pure sprint-type events, and the relevant kinematic results were mainly descriptive overall, this comparison is no longer presented as a figure in the main text, to avoid misinterpretation of a descriptive cross-study comparison as a formal mechanism-stratified statistical comparison. Therefore, this part of the results is considered only a hypothesis-generating observation and not a basis for the main inference.

#### 3.6.2 Sex comparison

The sex comparison results did not show a difference pattern with a consistent direction. Because the female sample was mainly derived from a single video-analysis study of women’s football, whereas the male results were derived from pooled or reference estimates from multiple male studies, this comparison was essentially a descriptive cross-study contrast rather than a strict within-study test of sex effects. Exploratory comparisons based on the positional relationship with the 95% prediction intervals from the male main analyses showed that the female single-study estimates and their 95% CIs for most core epidemiological indicators were within or partially overlapped with the male 95% prediction intervals, providing no indication of a stable direction of sex differences (20, 35, 36). To avoid implying a formal between-sex statistical comparison, these epidemiological comparisons are described in the text and presented only in Supplementary Figure S5 rather than as a main-text figure.

**Figure 3:**
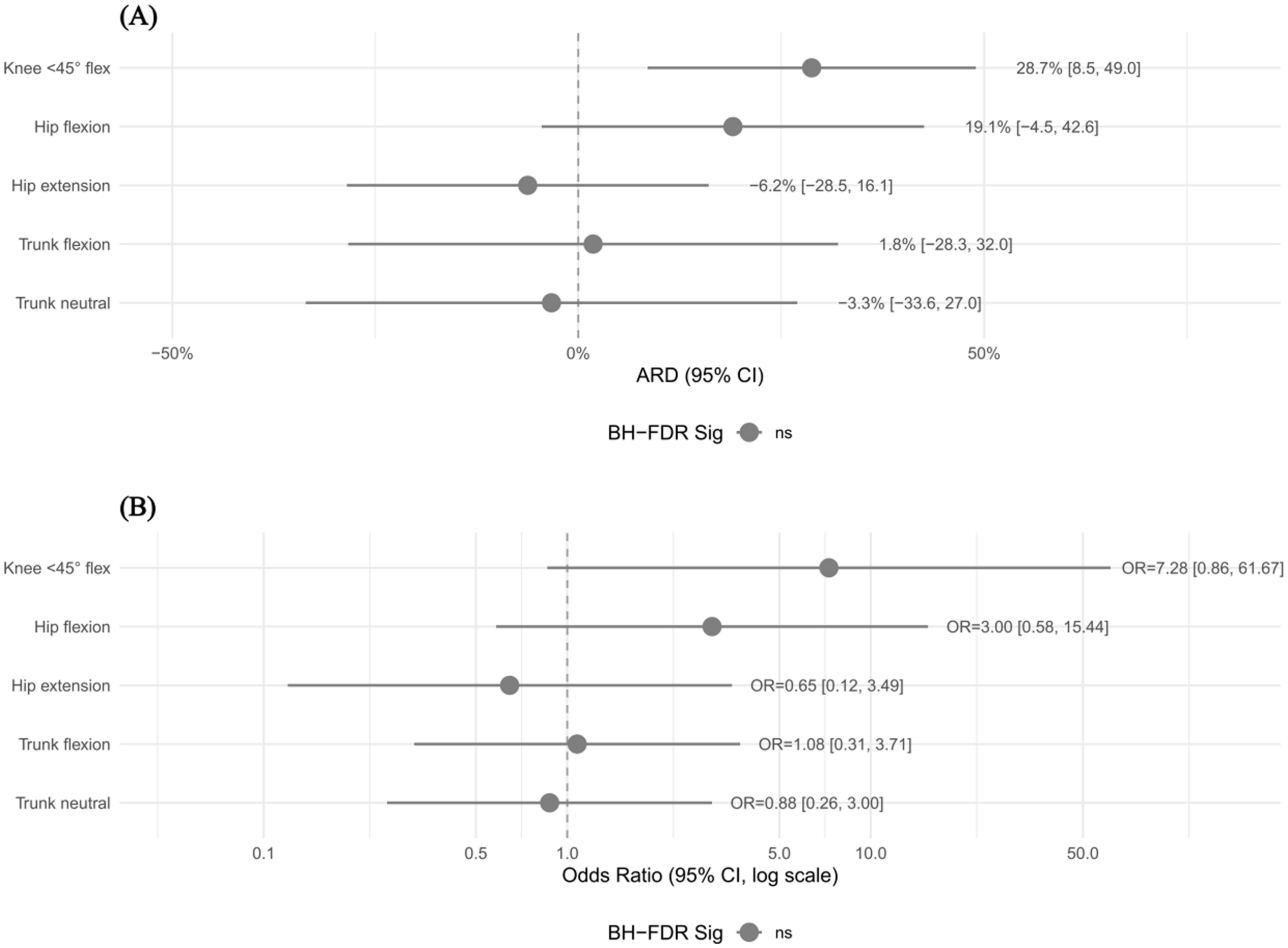
Exploratory kinematic comparison between male reference estimates and the female single-study estimate. Panel **(A)** presents absolute risk differences (ARDs) with 95% confidence intervals (CIs), calculated as the male reference estimate minus the female single-study estimate. Panel **(B)** presents the corresponding odds ratios (ORs) on a logarithmic scale, where OR > 1 indicates a higher proportion in the male reference sample. These estimates are exploratory cross-study contrasts for the same kinematic variables and are not confirmatory estimates of sex effects. They do not compare male sprint-type cases with female mixed-type cases or any other different mechanism categories.

Exploratory comparisons of discrete kinematic variables are shown in Figure 3. The ARD and OR in Figure 3 both represent descriptive cross-study contrasts between male reference estimates and the female single-study estimate for the same kinematic variable; they do not compare male sprint-type cases with female mixed-type cases, nor do they estimate a within-study sex effect. For discrete kinematic variables, Fisher’s exact test showed that none of the 5 prespecified variables reached statistical significance after Benjamini–Hochberg false discovery rate correction (22). Among them, the absolute risk difference for knee flexion <45° was relatively large (ARD = 28.7, 95% CI 8.5, 49.0%), but this result was relatively sensitive to angle binning and imputation assumptions. Given that this part of the analysis was based on contrasts between female single-study estimates and male pooled or reference estimates, the relevant results are suitable only for hypothesis generation in subsequent studies and should not be interpreted as confirmatory estimates of sex effects (20, 35–37).

### 3.7 Sensitivity analyses

The sensitivity analyses mainly comprised two parts: scenario analyses for recoding knee joint angles and leave-one-out (LOO) analyses. To address the inconsistency in knee joint angle binning, three recoding scenarios, namely Min, Uniform, and Max, were specified in this study. The results showed that, under different recoding schemes, the direction of the exploratory contrast for “knee flexion <45°” between the male reference estimate and the female single-study estimate was consistent, and the ARD estimates and Fisher’s exact test results were generally similar. For variables with *I*^2^ > 75%, the LOO analyses showed that Gandarias-Madariaga et al. 2025 was one of the main influential studies for running-related action, with-ball status, without-ball status, non-contact injury, and sprint-type, stretch-type, and mixed-type proportions; however, after exclusion of this study, changes in most pooled estimates were limited, and the direction of the main results did not change (38, 39). Given that different video-analysis studies were not entirely consistent in their mechanism classification frameworks and situational categorization, this finding is more appropriately interpreted as an indication of potential sources of heterogeneity rather than as a basis for definitive attribution to a single study (6, 8, 39).

### 3.8 Supplementary analyses

The supplementary OR analyses showed that the comparison of with ball vs. without ball had high heterogeneity and was noticeably influenced by a single study; therefore, the related results were retained only in the Supplementary materials and were not used for the main inferential conclusions (38, 39). The all-sample OR results after inclusion of the female sample were generally consistent in direction with the male primary analyses and were therefore also retained in the Supplementary Materials as supplementary results to further examine the consistency of the main analytical direction. Given that the current video-analysis evidence on HSI in women’s football remains limited, these results are presented for supplementary purposes only (6, 8). Funnel plots were presented only as supplementary visual assessments. Because fewer than 10 studies were available for each comparison, both visual inspection and formal asymmetry testing were considered underpowered and potentially misleading. Therefore, no formal inference regarding small-study effects or publication bias was made (23). The remaining supplementary OR comparisons and funnel-plot results are shown in Supplementary Figures S1, S2, and S9.

## 4 Discussion

### 4.1 Main findings

Among the five prespecified pairwise comparisons, higher relative reporting frequencies were observed only for non-contact versus indirect contact (OR = 2.51) and sprint-type versus mixed-type (OR = 2.01). The other three primary comparisons—sprint-type versus stretch-type, offensive versus defensive, and first half versus second half—did not show clear differences. These results are consistent with the predominance of non-contact HSI across multiple team sports and accord with the biomechanical model in which the hamstrings sustain peak eccentric loading during the late swing phase of high-speed running (5, 8). By contrast, sprint vs. stretch, offensive vs. defensive, and first half vs. second half showed no clear differences, suggesting that the distribution of injury-inciting scenarios across tactical and temporal dimensions may be more dispersed than previously expected. Single-arm proportion pooling further showed that sprint-type (48.8%), running-related action (86.6%), and non-contact (89.4%) accounted for relatively high proportions in the overall distribution, although these estimates showed substantial heterogeneity and are more appropriately interpreted as descriptive background. It should be noted that the ORs in this study reflect the relative reporting advantage of different event types within the same analytical dimension, rather than population risk ratios, and are therefore more suitable for characterizing relative patterns in the current video-based evidence (40).

### 4.2 Comparison with previous research and mechanistic interpretation

No clear difference was observed for sprint vs. stretch (OR = 1.64, *I*^2^ = 84%), and heterogeneity was high. From a biomechanical perspective, sprint-type HSI typically occurs at the moment of peak eccentric loading during the late swing phase, whereas stretch-type injury more often involves passive excessive lengthening during kicking, sliding tackles, or overstretching actions (8). These two mechanisms differ fundamentally in their kinetic characteristics, but the current video-based studies have not yet achieved full consistency in the operational definition and interpretive boundaries of stretch-type injuries, which may be an important source of the substantial heterogeneity in this comparison. Therefore, this finding of no clear difference should be interpreted more appropriately as reflecting the limitations imposed by differences in classification frameworks on quantitative synthesis, rather than indicating that the two types of mechanisms can be considered equivalent in terms of occurrence frequency (6, 8).

No clear differences were observed in either the tactical or temporal comparisons. The OR for offensive vs. defensive was close to 1 and *I*^2^ was 0%, indicating a similar distribution of HSI between offensive and defensive phases, with a consistent direction across studies. This finding is noteworthy because it differs from the defense-oriented situational pattern reported in some football ACL video-analysis studies. For example, a recent video-analysis study of male professional English soccer reported more ACL injuries during defensive than offensive playing situations, and identified pressing/tackling, being tackled, landing from a jump, and regaining balance after kicking as the main patterns among indirect-contact and non-contact ACL injuries (41). Similar situational patterns, including pressing/tackling and regaining balance after kicking, have also been reported in professional women’s soccer ACL injuries (42). In contrast, the present review observed a similar distribution of HSI between offensive and defensive situations, which may suggest that high-speed running-related eccentric loading and rapid braking demands during the running cycle can occur in both offensive and defensive phases. This cross-injury comparison should be interpreted only as contextual background, because the present review did not directly analyze ACL injuries. However, because the included video-analysis studies did not provide exposure-time denominators, such as the actual duration of high-speed running in different tactical phases, this result should be understood as a distributional feature of reported injury events and cannot be directly interpreted as a difference in the true risk of HSI across different tactical contexts. Regarding temporal distribution, no clear difference was observed for first half vs. second half; across the six 15-min periods divided as 0–15, 16–30, 31–45, 46–60, 61–75, and 76–90 min, single-arm proportions ranged only from 14.1 to 18.2%, showing no stable temporal clustering. This is not entirely consistent with descriptions from some professional football monitoring studies suggesting a higher HSI incidence toward the end of each half (43), but one possible explanation is that HSI occurrence is influenced by the interaction of multiple factors, including immediate mechanical load, cumulative neuromuscular fatigue, and prior exposure, rather than following a simple pattern of linear increase over match time (8, 43, 44).

The descriptive comparison of kinematic postures showed that sprint/mixed-related and stretch-related samples were directionally similar in knee posture, with flexion <45° predominating in both, whereas some differences were observed in hip and trunk alignment. This observation is partly consistent with theoretical expectations: in sprint-type actions, the hip is often in a flexed position to decelerate the limb during late swing, whereas in stretch-type actions the hip may reach greater flexion or abduction angles (5, 8). However, because the number of included studies was limited and one of the included studies did not involve a pure sprint-type sample, these differences cannot yet be regarded as strict evidence of mechanism stratification. The sex comparison likewise did not show differences that remained stably significant after Benjamini–Hochberg correction (22); knee flexion <45° showed the largest ARD (28.7%), but this result was sensitive to the assumptions used for angle imputation. Given that only one female study is currently available for comparison, the relevant effect sizes are more appropriately used for hypothesis generation and sample size estimation in future studies, rather than being interpreted as confirmatory sex-specific mechanistic differences (6, 45).

### 4.3 Practical implications

Based on the above findings, the implications of this study for football HSI prevention research and training-content design should be interpreted as hypotheses for future intervention design rather than as direct evidence supporting specific training priorities. Within this interpretive boundary, the findings may be considered from three perspectives. First, given that the reported frequency of HSI in non-contact contexts was higher than that in indirect-contact contexts, future studies may consider examining eccentric load tolerance during high-speed running, control during the terminal swing phase of running, and neuromuscular control during the single-leg support phase. Second, the similar distribution of HSI between offensive and defensive phases suggests that future studies of high-speed running-related training or monitoring may consider including different tactical contexts, rather than assuming that HSI is concentrated in a single match phase. Third, because the within-match temporal distribution did not show clear clustering, load-management-related research may need to examine cumulative high-speed exposure across the entire match, rather than assuming that HSI risk is concentrated only in the late stages of each half (43, 44).

It should be emphasized that the above practical implications still need to be interpreted in conjunction with independent evidence from preventive intervention studies. Previous systematic reviews have suggested that prevention programs centered on eccentric training are associated with lower HSI incidence, but methodological reappraisals have also indicated that uncertainty remains regarding the magnitude and certainty of this effect (46, 47). In addition, because heterogeneity was high in the sprint vs. stretch comparison, the current evidence remains insufficient to support differential preventive recommendations for different mechanistic backgrounds. This issue awaits further clarification in future research after greater standardization of mechanism definitions.

### 4.4 Strengths and limitations

The strengths of this study are as follows. First, the research question focused on the relatively homogeneous evidence source of football match video analysis, and five primary pairwise comparisons were prespecified, making the objective of the main analyses relatively clear. Second, the main analyses, single-arm proportion pooling, and exploratory analyses were distinguished at the level of results, thereby reducing the risk of misinterpreting descriptive findings as primary inferential conclusions. Third, for dynamic denominators, differences in knee-joint binning, and variables with high heterogeneity, supplementary diagnostics and sensitivity analyses were conducted to improve the transparency of result interpretation.

This study also has several limitations. First, the number of included studies was limited, and several primary comparisons showed substantial heterogeneity, indicating persistent differences across studies in mechanism definitions, match contexts, and video-interpretation standards. Second, the existing evidence is almost entirely derived from professional or elite football and is dominated by male samples, which limits the generalizability of the findings to female players and non-professional populations. In this study, female-related evidence was mainly derived from a single video-analysis study of women’s football, and the number of female cases, the number of analyzable videos, competition sources, and season coverage were not fully symmetrical with those of the male samples. Therefore, the sex-stratified results are suitable only for describing potential trends and generating hypotheses for subsequent studies, and cannot be interpreted as confirmed sex-specific differences in HSI mechanisms. In particular, it should be emphasized that the single source of evidence from women’s football prevents this study from distinguishing the relative contributions of true sex differences, differences in study sources, and differences in video availability to the results. Third, all studies were based on two-dimensional match video, and joint-angle estimation and action classification remain subject to measurement error because of limitations in camera angle, frame rate, and image completeness. Fourth, reporting was not fully consistent across studies with respect to mechanism definitions, time binning, and posture classification. Although this study attempted to reduce these effects through dimension-specific denominators and sensitivity analyses, their influence on the pooled results could not be completely eliminated. Overall, the current findings are more appropriately understood as relative reporting patterns within the available video-based evidence, rather than as high-certainty mechanistic conclusions (6, 39).

## 5 Conclusion

This meta-analysis integrated 342 HSI cases from 7 video-analysis studies of football matches and found higher reported frequencies for non-contact versus indirect contact and for sprint-type versus mixed-type; however, no stable differences were observed between offensive/defensive phases, first/second halves, or sprint/stretch. These findings may provide mechanistic hypotheses for prevention research and training-content design, particularly for examining non-contact injury scenarios in high-speed running contexts and related eccentric load adaptations; however, because this study was based on video-analysis evidence, the preventive effects of specific training strategies cannot be directly inferred. Because the number of included studies was limited, mechanism definitions have not yet been standardized, and video evidence in female players is insufficient, future studies should further improve the standardization of video-analysis reporting and accumulate larger, consistently defined cross-sex samples to enhance the generalizability of the evidence.

## Data Availability

The original contributions presented in the study are included in the article/Supplementary material, further inquiries can be directed to the corresponding authors.
