# Supplementary material for: Mechanisms, injury patterns, and kinematic features of hamstring strain injuries in football (soccer): a systematic review and meta-analysis of video-analysis studies
Source: Front Public Health. 2026 May 21;14:1846524. doi: 10.3389/fpubh.2026.1846524 (PMC13233188; doi:10.3389/fpubh.2026.1846524)
Supplement: Supplementary file 1 [file Supplementary_file_1.DOCX]

**Supplementary Table S1. Item-level JBI risk-of-bias assessment of the included studies**

| **Study**  **(First Author, Year)** | **Q1** | **Q2** | **Q3** | **Q4** | **Q5** | **Q6** | **Q7** | **Q8** | **Q9** | **Q10** | **Total Score**  **(/10)** | **Risk-of-Bias Grade** |
| --- | --- | --- | --- | --- | --- | --- | --- | --- | --- | --- | --- | --- |
| Pellegrini 2025 | 1 | 1 | 1 | — | — | — | — | 1 | — | 1 | 5 | Moderate Risk |
| Jokela 2023 | 1 | 1 | 1 | — | — | — | — | 1 | — | 1 | 5 | Moderate Risk |
| Vermeulen 2024 | 1 | 1 | 1 | — | — | — | — | 1 | — | — | 4 | High Risk |
| Della Villa 2023 | 1 | 1 | 1 | — | — | — | — | 1 | — | 1 | 5 | Moderate Risk |
| Gandarias-Madariaga et al. 2025 | 1 | 1 | 1 | — | — | — | — | 1 | — | — | 4 | High Risk |
| Gronwald et al. 2022 | 1 | 1 | 1 | — | — | — | — | 1 | — | — | 4 | High Risk |
| Aiello et al. 2023 | 1 | 1 | 1 | — | — | — | — | 1 | — | — | 4 | High Risk |

Note: Each item was scored as Yes = 1 or No/Unclear/Not applicable = 0 for the purpose of the present within-review appraisal. In the item columns, “—” indicates No/Unclear/Not applicable (=0). Only item-level scores are presented in this table; no separate evidence column is displayed. Total scores range from 0 to 10. The grading thresholds reported here were predefined operational rules for within-review comparison only and should not be interpreted as official JBI cut-offs.

JBI Case Series items: Q1 = Were there clear criteria for inclusion in the case series? Q2 = Was the condition measured in a standard, reliable way for all participants included in the case series? Q3 = Were valid methods used for identification of the condition for all participants included in the case series? Q4 = Did the case series have consecutive inclusion of participants? Q5 = Did the case series have complete inclusion of participants? Q6 = Was there clear reporting of the demographics of the participants in the study? Q7 = Was there clear reporting of clinical information of the participants? Q8 = Were the outcomes or follow-up results of cases clearly reported? Q9 = Was there clear reporting of the presenting site(s)/clinic(s) demographic information? Q10 = Was statistical analysis appropriate? Item descriptions are based on the JBI Critical Appraisal Checklist for Case Series[12].

**Supplementary Table S2. Item-level reporting-completeness assessment based on core STROBE-SIIS items**

| **Study**  **(First Author, Year)** | **S1** | **S2** | **S3** | **S4** | **S5** | **S6** | **S7** | **S8** | **Total Score**  **(8)** | **Percentage**  **(%)** | **Reporting Completeness Grade** |
| --- | --- | --- | --- | --- | --- | --- | --- | --- | --- | --- | --- |
| Pellegrini 2025 | 1 | 0.5 | 1 | 1 | 1 | 0.5 | 0.5 | 1 | 6.5 | 81.2 | Complete |
| Jokela 2023 | 1 | 0.5 | 1 | 1 | 1 | 0.5 | — | 1 | 6 | 75 | Moderate completeness |
| Vermeulen 2024 | 1 | 0.5 | 1 | 1 | 1 | 0.5 | — | 0.5 | 5.5 | 68.8 | Moderate completeness |
| Della Villa 2023 | 1 | 0.5 | 1 | 1 | 1 | 0.5 | — | 0.5 | 5.5 | 68.8 | Moderate completeness |
| Gandarias-Madariaga et al. 2025 | 1 | 0.5 | 1 | 1 | 1 | 0.5 | — | 1 | 6 | 75 | Moderate completeness |
| Gronwald et al. 2022 | 1 | 0.5 | 1 | 1 | 1 | 0.5 | 0.5 | 1 | 6.5 | 81.2 | Complete |
| Aiello et al. 2023 | 1 | 0.5 | 1 | 1 | 1 | 0.5 | 0.5 | 1 | 6.5 | 81.2 | Complete |

Note: Each item was scored as Present = 1, Partially reported = 0.5, or Absent/NA = 0. Only item-level scores are presented in this table. Percentage values are expressed on a 0–100 scale and were calculated as (total score / 8) × 100. The grading thresholds reported here were predefined operational rules for within-review comparison only.

STROBE-SIIS items: S1 = Study design described; S2 = Setting (competition level, league) reported; S3 = Participants (inclusion/exclusion criteria) described; S4 = Data sources/measurement methods described; S5 = Study size reported; S6 = Outcome definition (injury definition and diagnostic method) stated; S7 = Statistical methods described; S8 = Main results presented with appropriate measures. Items were adapted from the STROBE-SIIS extension[15] and selected as core reporting elements for this review.

**Supplementary Table S3. Item-level QA-SIVAS methodological-quality assessment of the included video-analysis studies**

| **Study (First Author, Year)** | **V1** | **V2** | **V3** | **V4** | **V5** | **V6** | **V7** | **V8** | **V9** | **V10** | **V11** | **V12** | **V13** | **V14** | **V15** | **V16** | **V17** | **V18** | **QA_Total** | **QA_Percent** | **QA_Quality** |
| --- | --- | --- | --- | --- | --- | --- | --- | --- | --- | --- | --- | --- | --- | --- | --- | --- | --- | --- | --- | --- | --- |
| Pellegrini 2025 | 1 | 1 | 1 | 1 | 1 | 1 | 0 | 1 | 0 | 0 | 0 | 1 | 1 | 1 | 1 | 1 | 1 | 1 | 14 | 77.8 | Good Quality |
| Jokela 2023 | 1 | 1 | 1 | 1 | 1 | 1 | 1 | 1 | 1 | 0 | 0 | 1 | 1 | 1 | 0 | 1 | 1 | 1 | 15 | 83.3 | High Quality |
| Vermeulen 2024 | 1 | 1 | 1 | 1 | 1 | 1 | 0 | 1 | 1 | 0 | 0 | 1 | 1 | 1 | 1 | 1 | 1 | 1 | 15 | 83.3 | High Quality |
| Della Villa 2023 | 1 | 1 | 1 | 1 | 1 | 1 | 0 | 1 | 1 | 0 | 0 | 1 | 1 | 1 | 1 | 1 | 1 | 1 | 15 | 83.3 | High Quality |
| Gandarias-Madariaga et al. 2025 | 1 | 1 | 1 | 0 | 0 | 1 | 0 | 0 | 1 | 0 | 0 | 1 | 1 | 1 | 1 | 1 | 1 | 1 | 12 | 66.7 | Moderate Quality |
| Gronwald et al. 2022 | 1 | 1 | 1 | 1 | 1 | 1 | 1 | 0 | 1 | 0 | 1 | 1 | 1 | 1 | 1 | 1 | 1 | 1 | 16 | 88.9 | High Quality |
| Aiello et al. 2023 | 1 | 1 | 1 | 1 | 1 | 1 | 1 | 0 | 0 | 0 | 1 | 1 | 1 | 1 | 1 | 1 | 1 | 1 | 15 | 83.3 | High Quality |

Note: Each item was scored as Yes = 1 or No/Not stated = 0. Only item-level scores are presented in this table. Percentage values are expressed on a 0–100 scale and were calculated as (total score / 18) × 100. The grading thresholds reported here were predefined operational rules for within-review comparison only.

**Supplementary Table S4. Video-analysis implementation characteristics of the included studies**

| **Study (First Author, Year)** | **Videos (A/D)** | **Raters, n** | **2D video analysis tool/platform** |
| --- | --- | --- | --- |
| Pellegrini, 2025 | 57/52 | 3 | Kinovea |
| Jokela, 2023 | 14/NR | 4 | QuickTime |
| Vermeulen, 2024 | 63/68 | 9 | VLC |
| Della Villa, 2023 | 103/18 | 3 | Kinovea |
| Gandarias, 2025 | 78/47 | 2 | Mediacoach |
| Gronwald, 2022 | 52/56 | 3 | Consensus form |
| Aiello, 2023 | 17/NR | 3 | FIIC |

Note: Videos (A/D) = numbers of videos analysed/discarded; NR = not reported. All included studies were based on two-dimensional match-video analysis. Della Villa, 2023 initially reported 103 lower-limb injury videos, of which 61 were confirmed HSI cases and were included in this review. Aiello, 2023 reported data from three consecutive seasons without specifying the calendar years.

**Supplementary Figure S1. Additional forest plots for six pairwise OR comparisons**


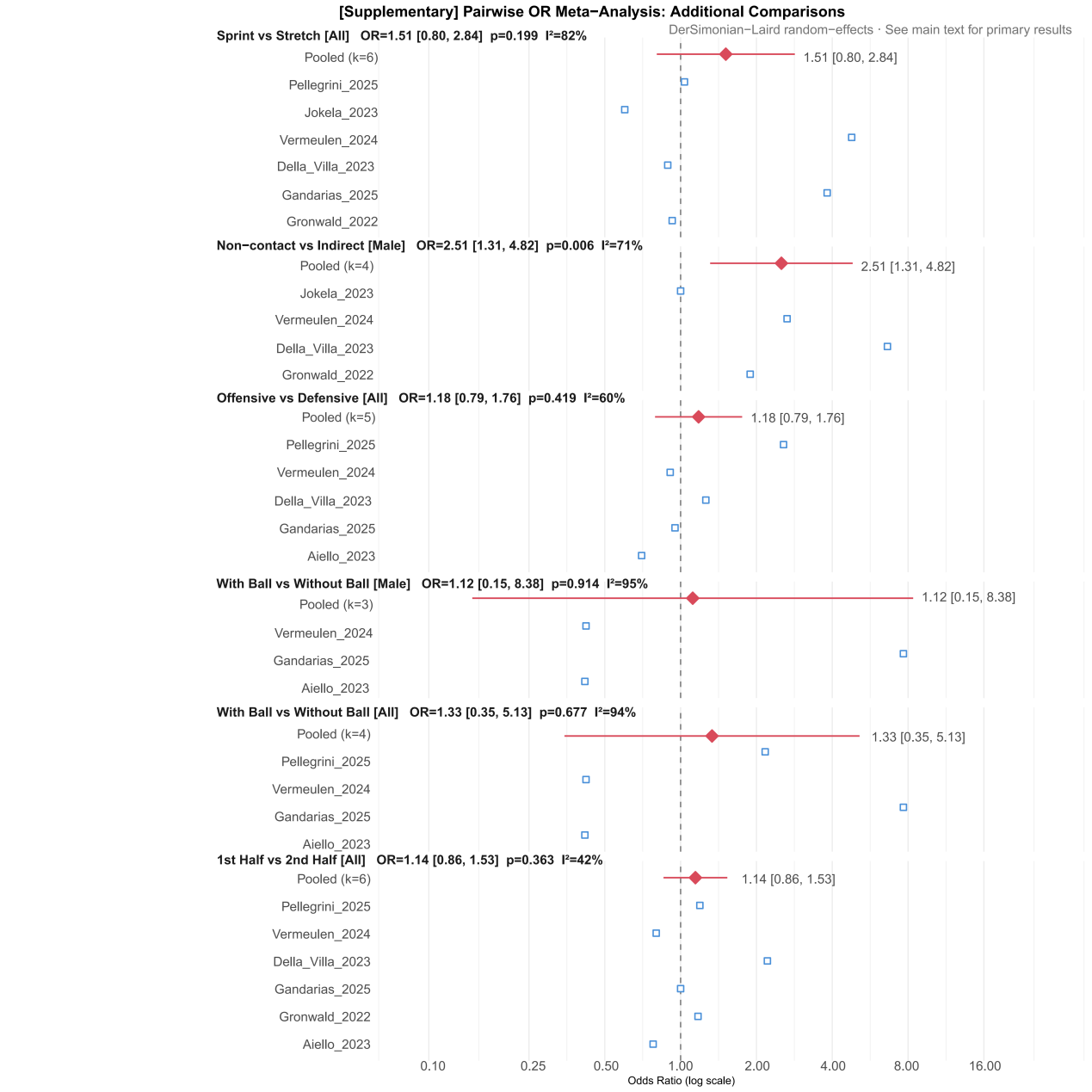


Note: Squares represent study-specific ORs, with square size proportional to study weight; diamonds represent pooled random-effects estimates; horizontal lines indicate 95% CIs; and the vertical dashed line indicates the null value (OR = 1.0). Pooled estimates were calculated using the DerSimonian–Laird method. Each comparison also reports the number of included studies (k), pooled OR, 95% CI, p value, and I².

**Supplementary Figure S2. Forest plots for all 11 pairwise OR comparisons**


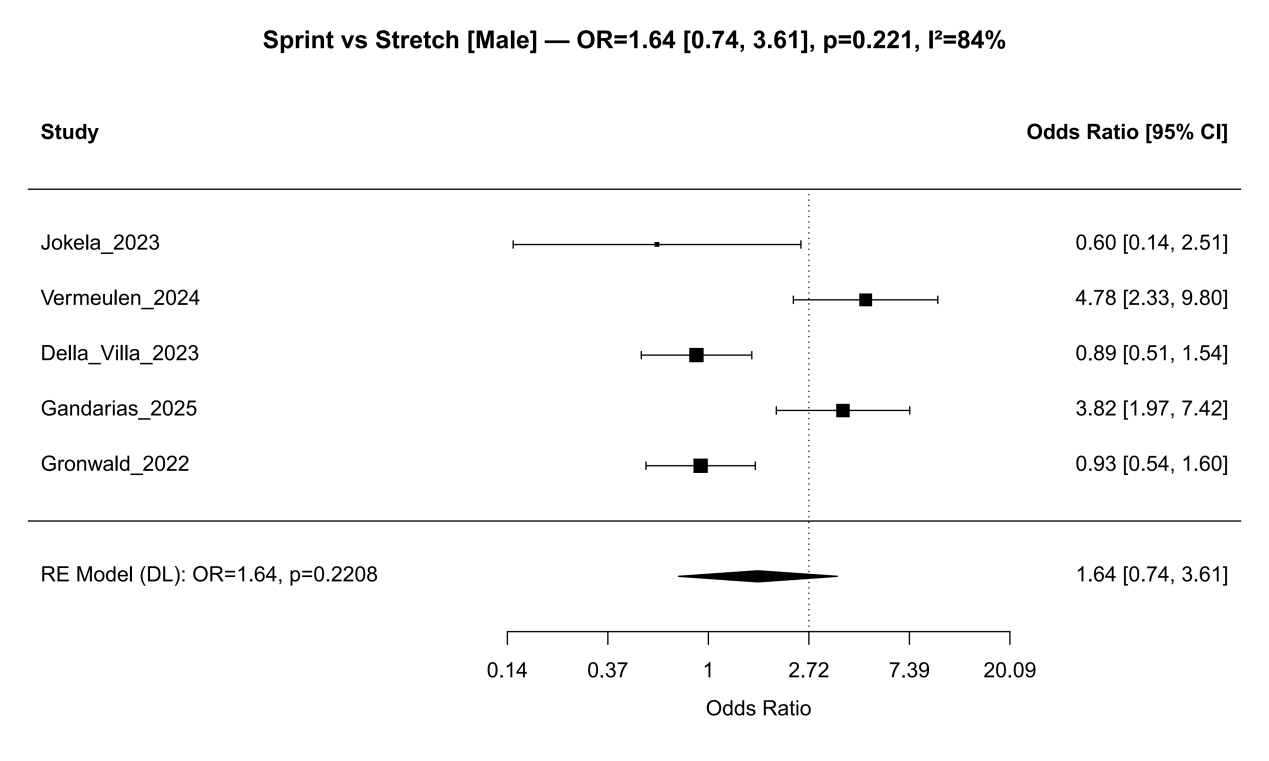


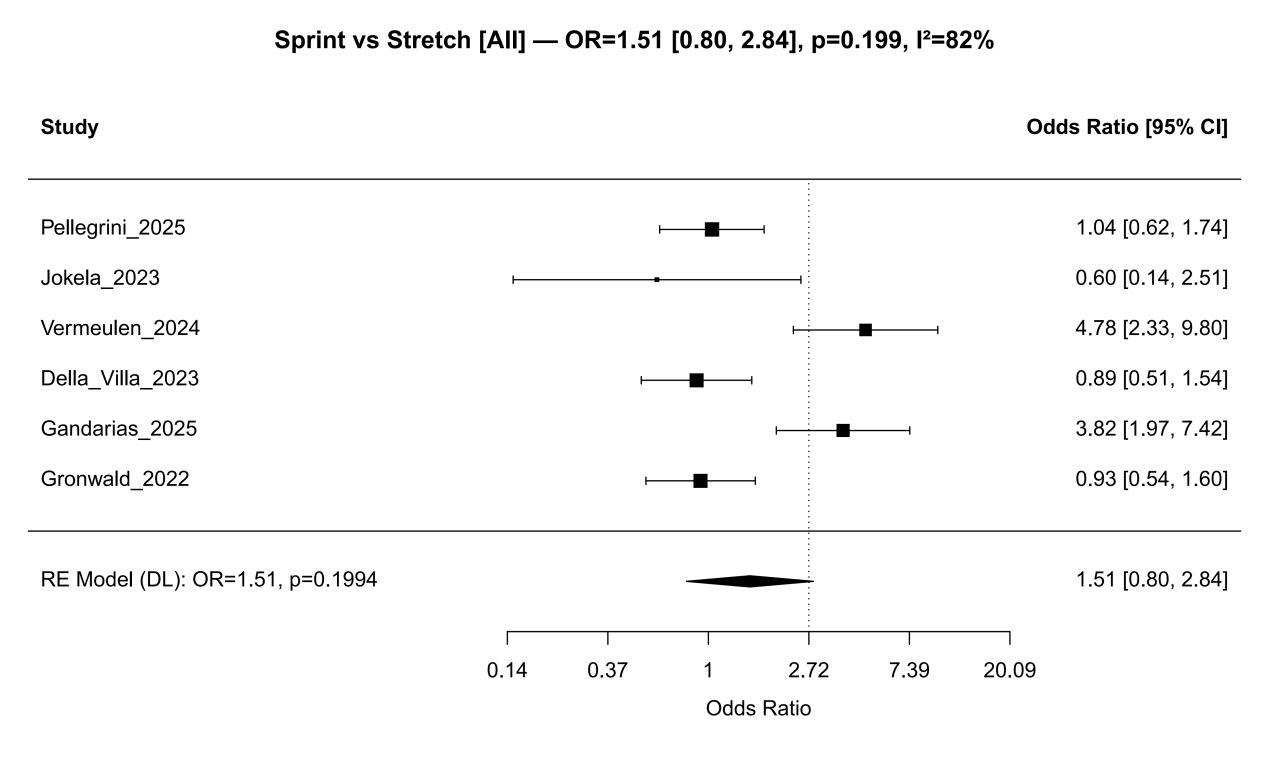


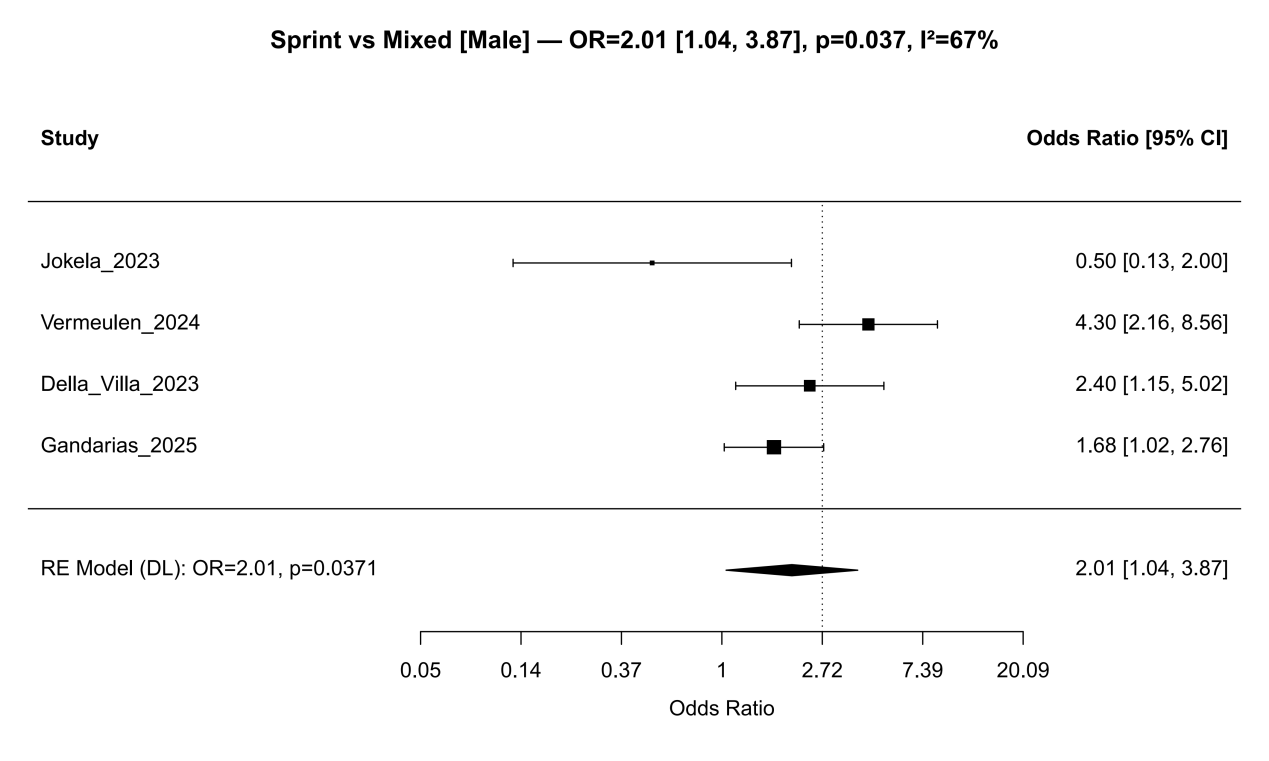


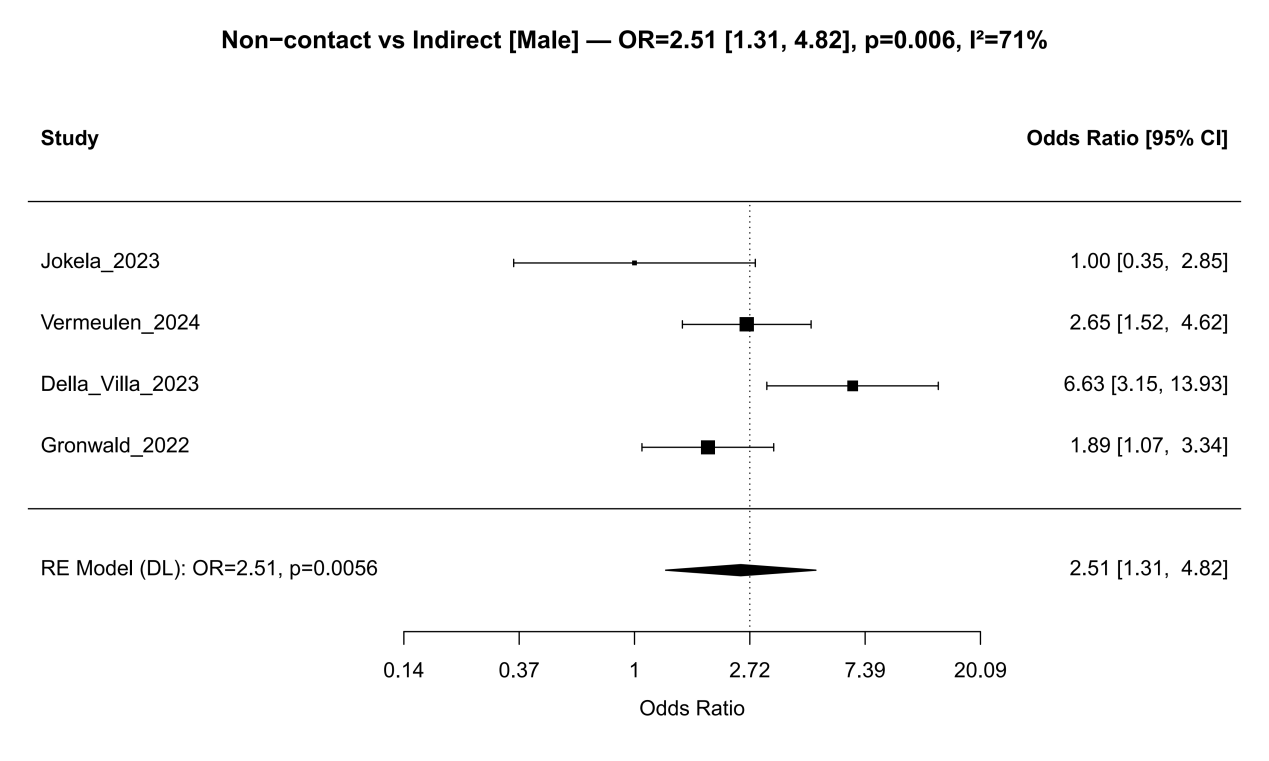


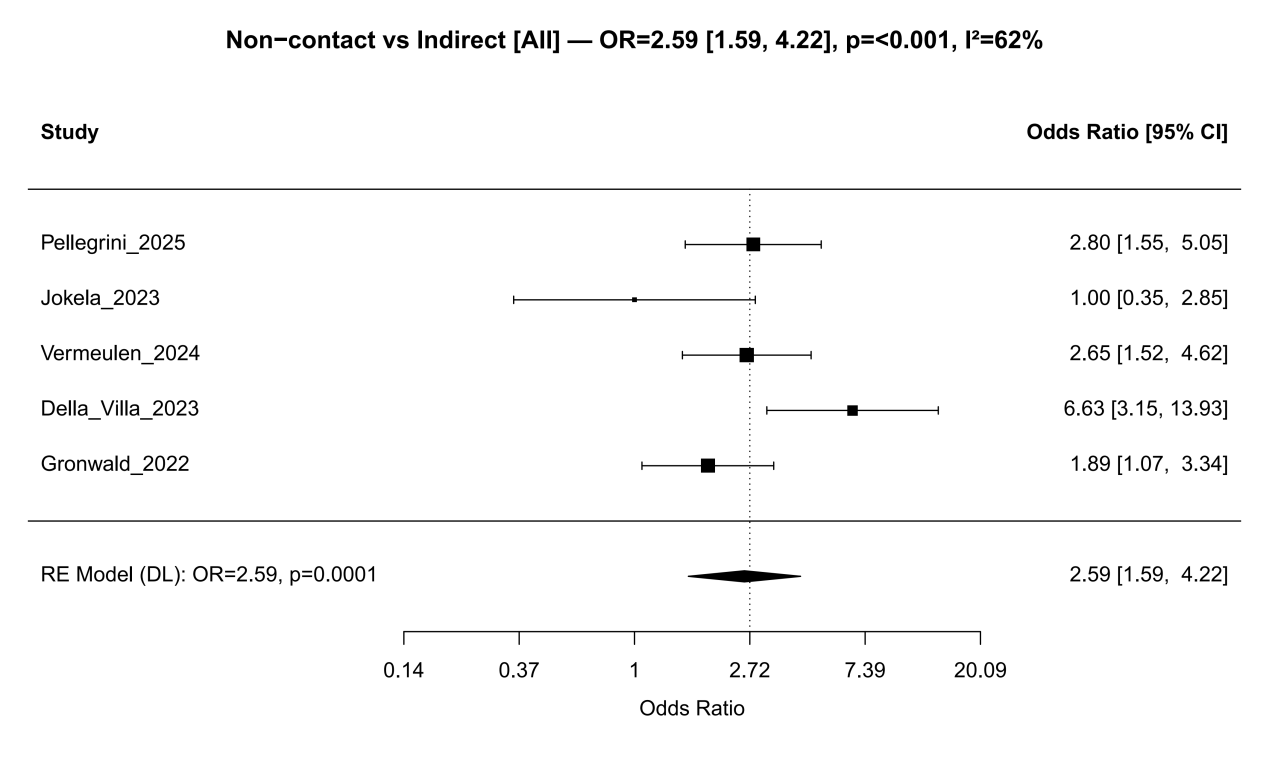


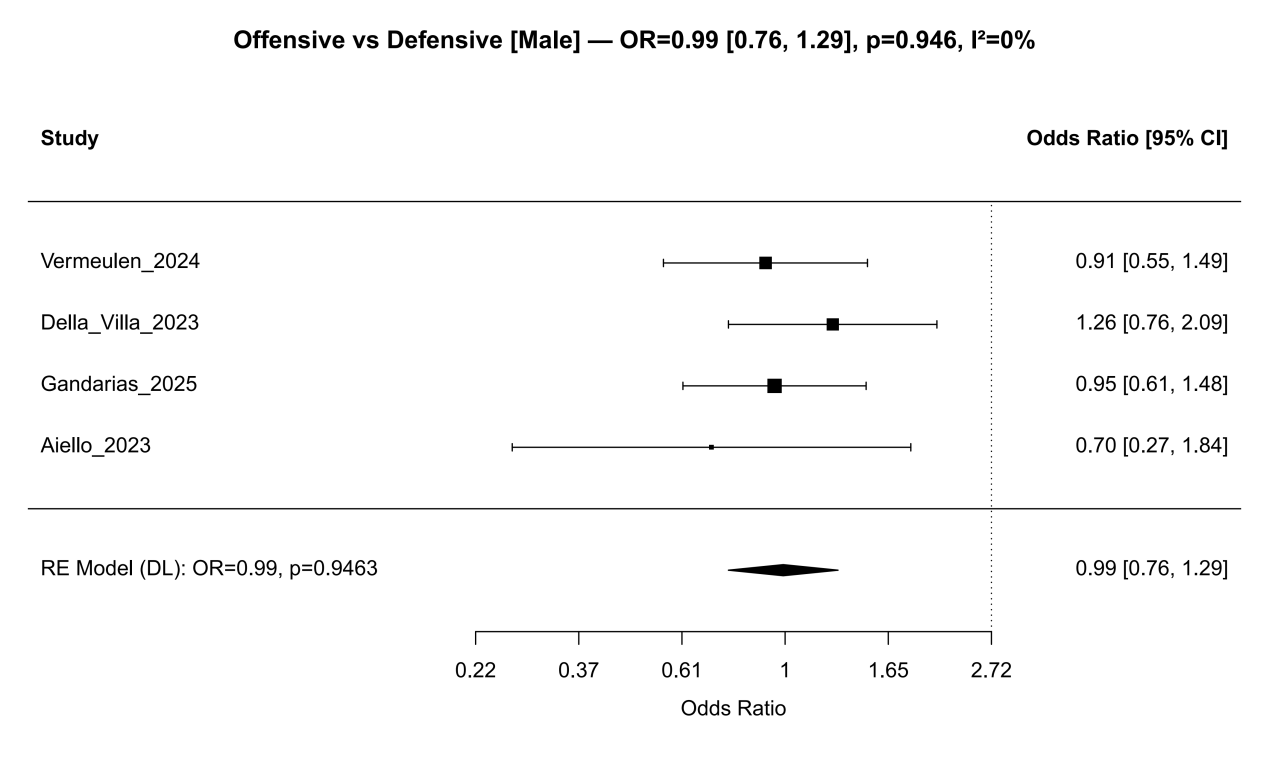


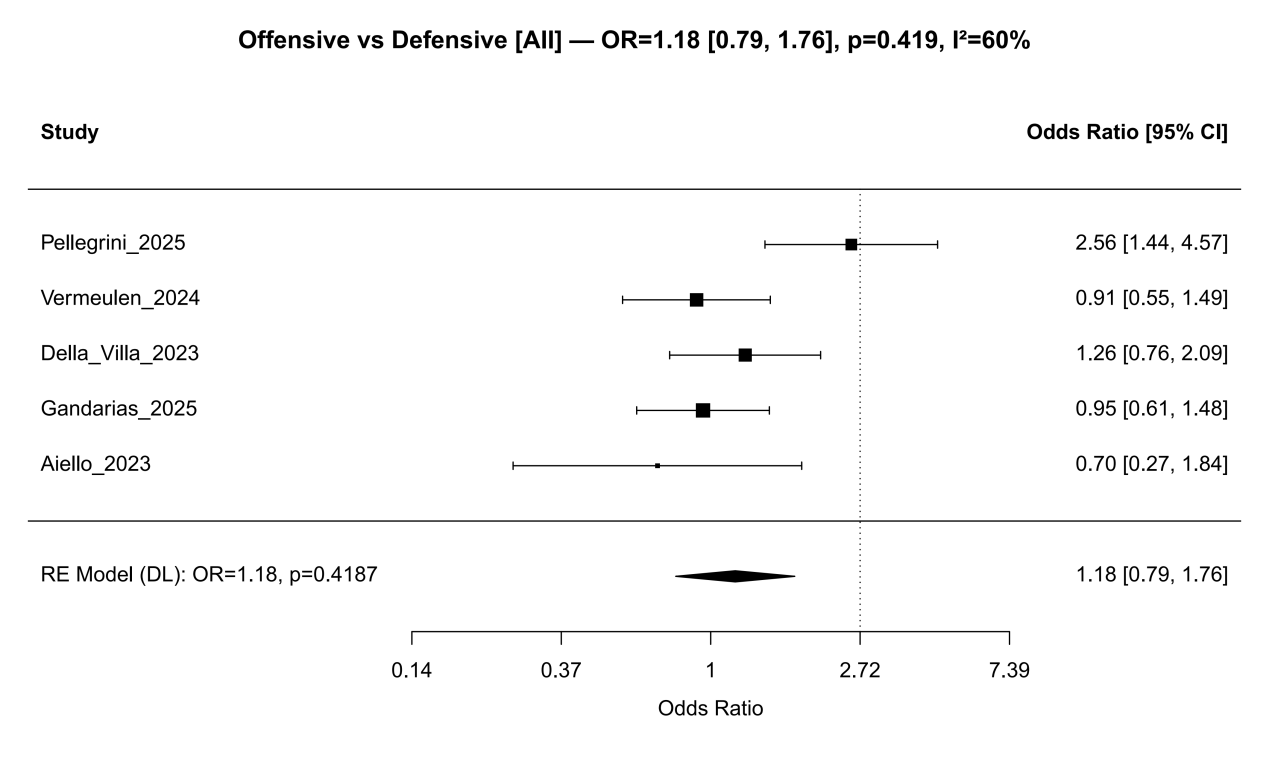


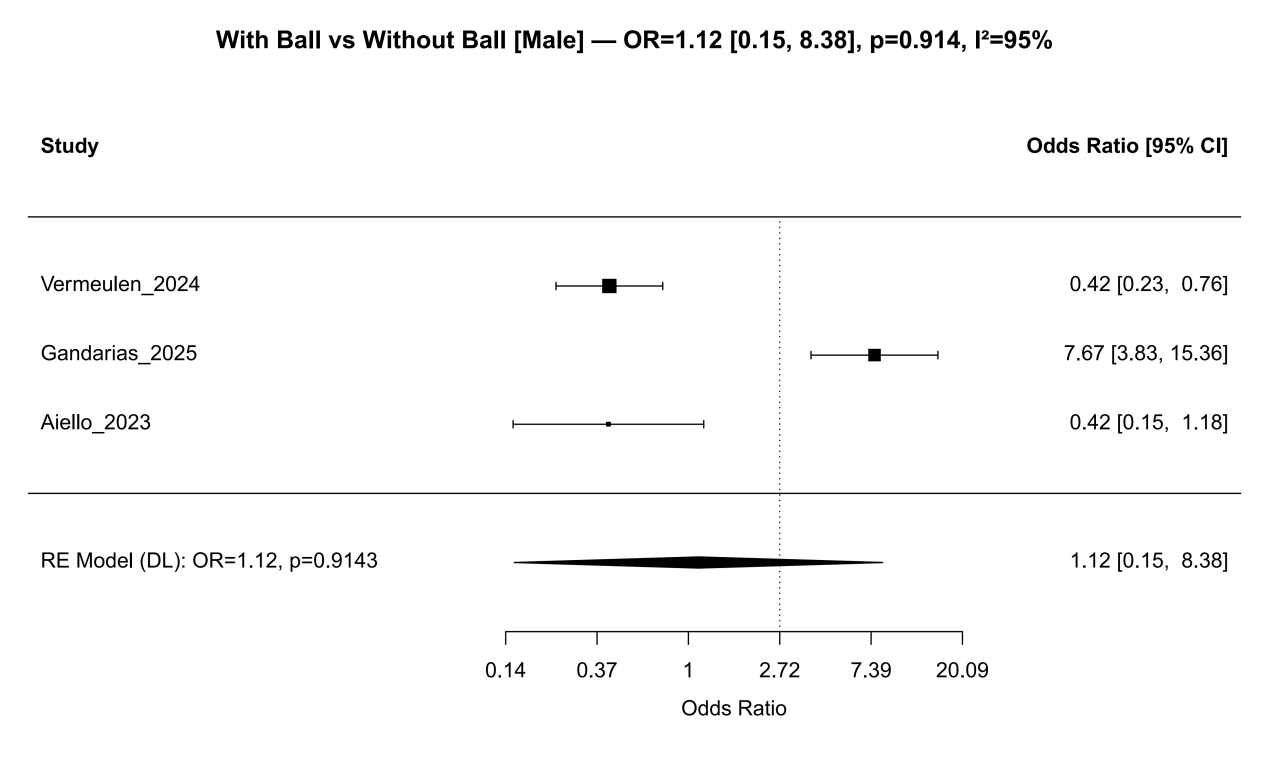


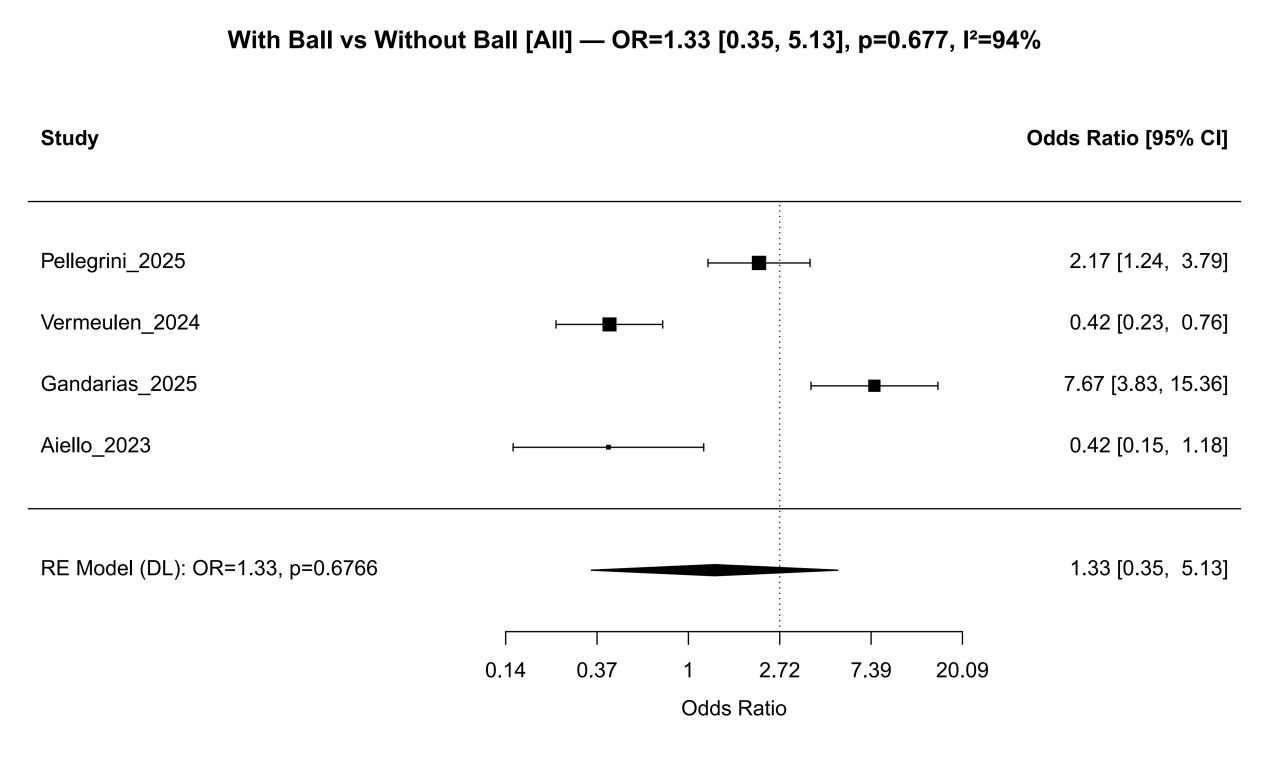


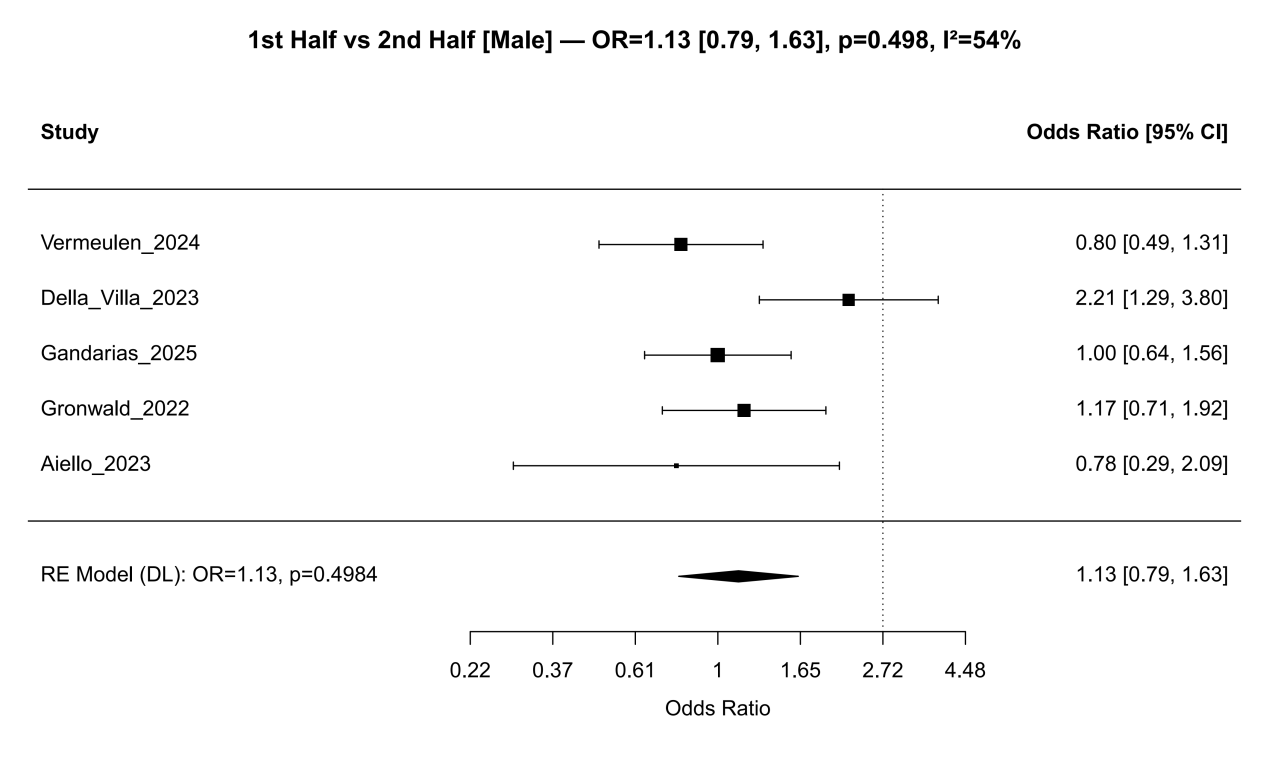


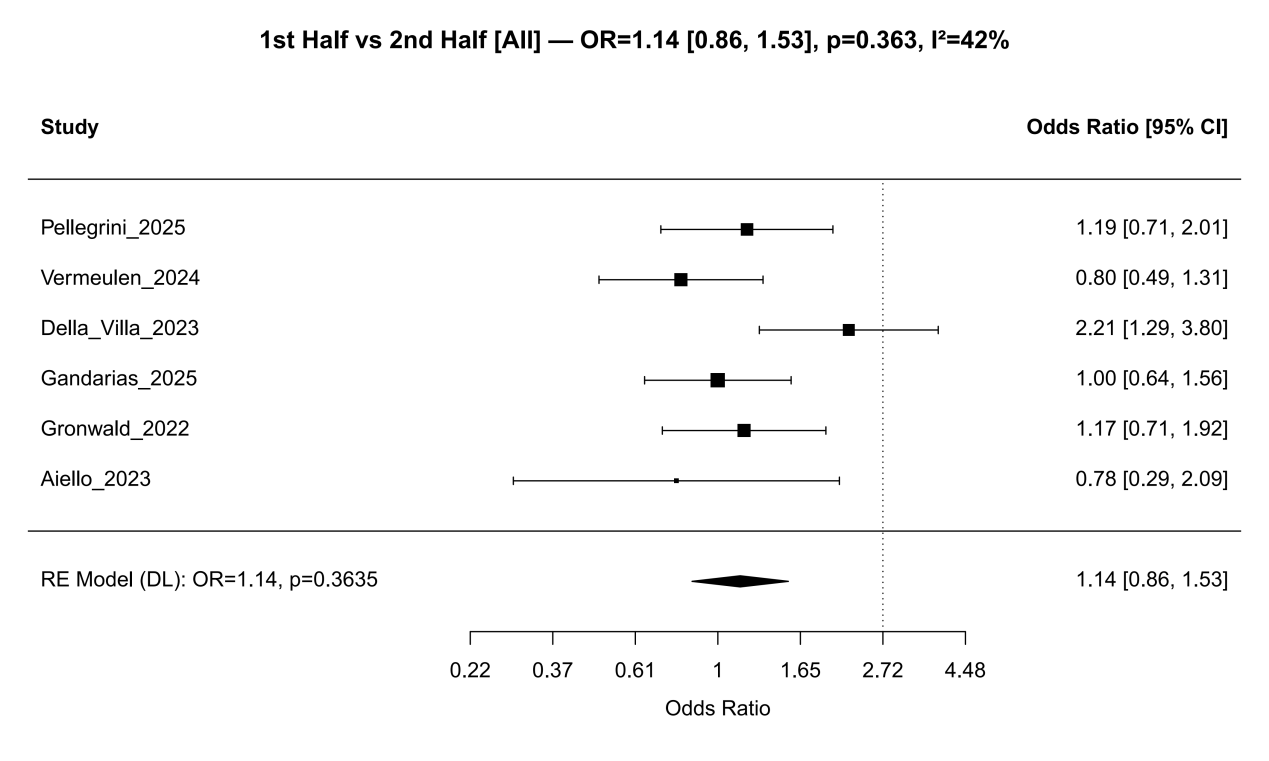


Note: Squares represent study-specific ORs, with area proportional to study weight; horizontal lines indicate 95% CIs; diamonds represent pooled estimates; and the vertical dashed line indicates the null value (OR = 1.0). Pooled estimates were calculated using the DerSimonian–Laird method, and heterogeneity is reported as I².

**Supplementary Figure S3. Dynamic-denominator diagnostic matrix**


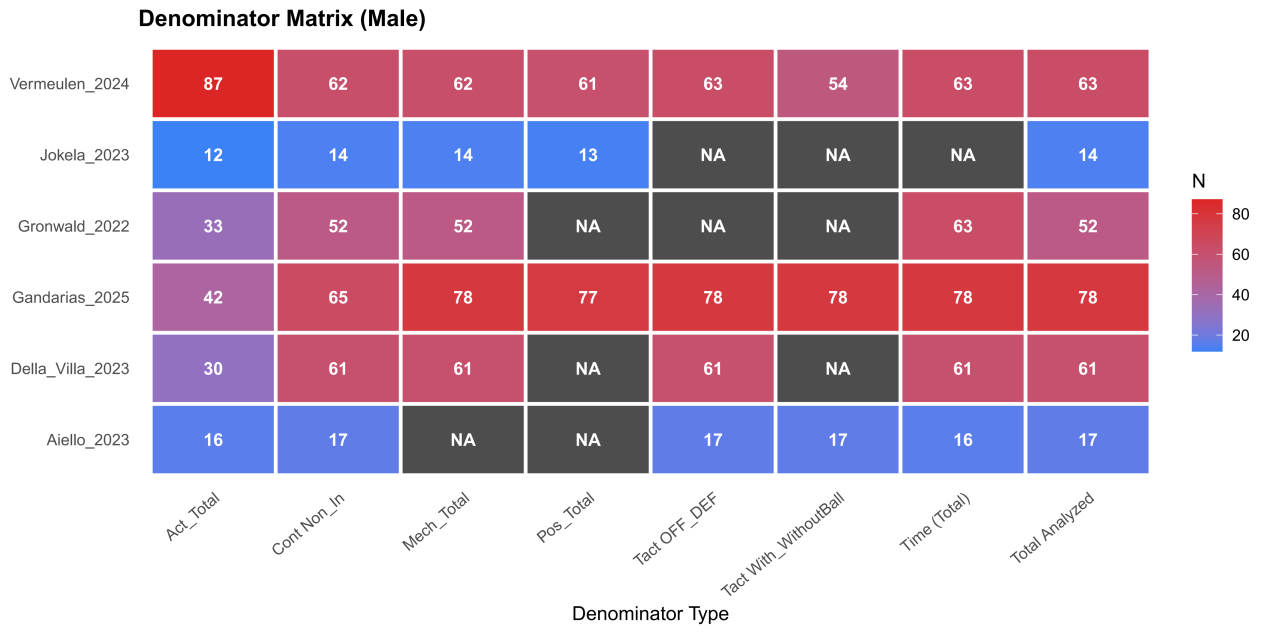


Note: This figure summarises denominator values across male-sample studies for the prespecified epidemiological comparison domains. Rows correspond to studies and columns correspond to denominator types. NA indicates that the denominator was either not reported in the original study or not applicable to the comparison. This figure is intended to assess denominator consistency and combinability before pairwise OR meta-analysis.

**Supplementary Figure S4. Position-by-mechanism heat map**


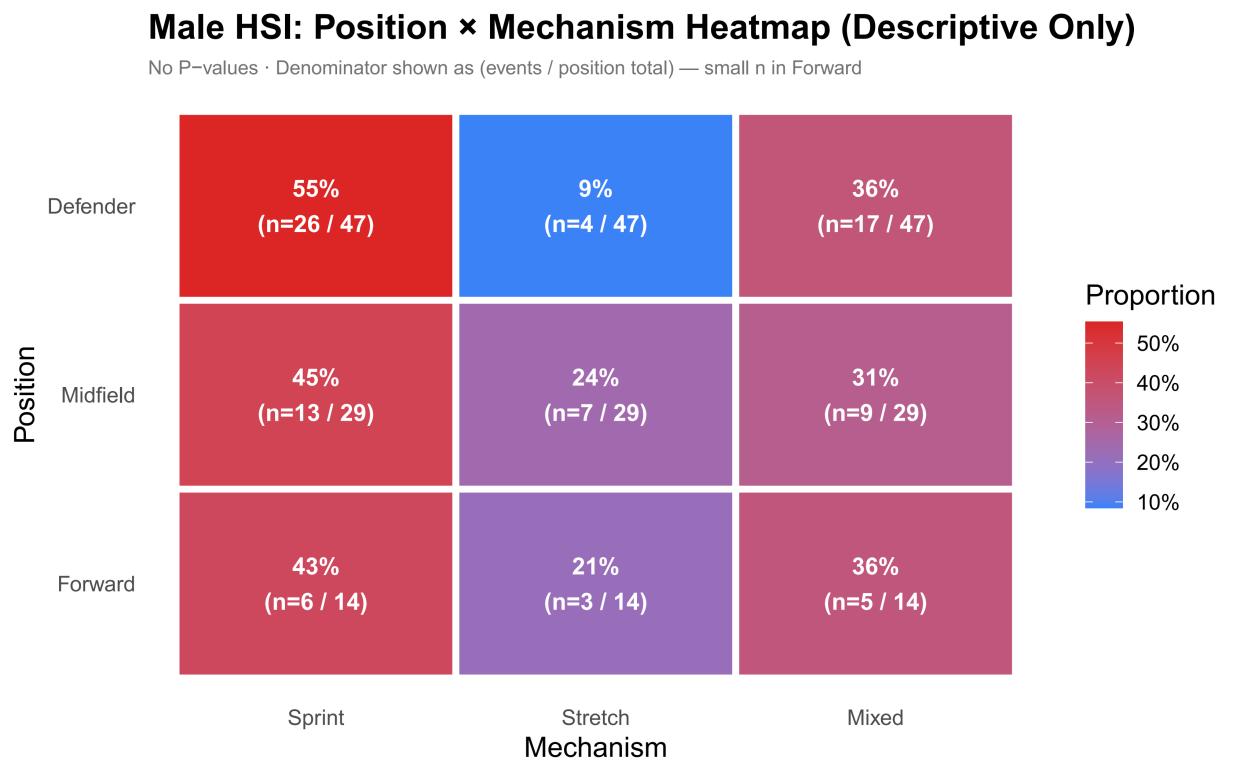


Note: Values are presented as percentages with corresponding counts [n/position total], with percentages calculated using the position-specific total as the denominator. This figure is descriptive only and is intended to illustrate the distribution of sprint-type, stretch-type, and mixed-type mechanisms across defenders, midfielders, and forwards.

**Supplementary Figure S5. Descriptive location of female single-study estimates relative to male prediction intervals**


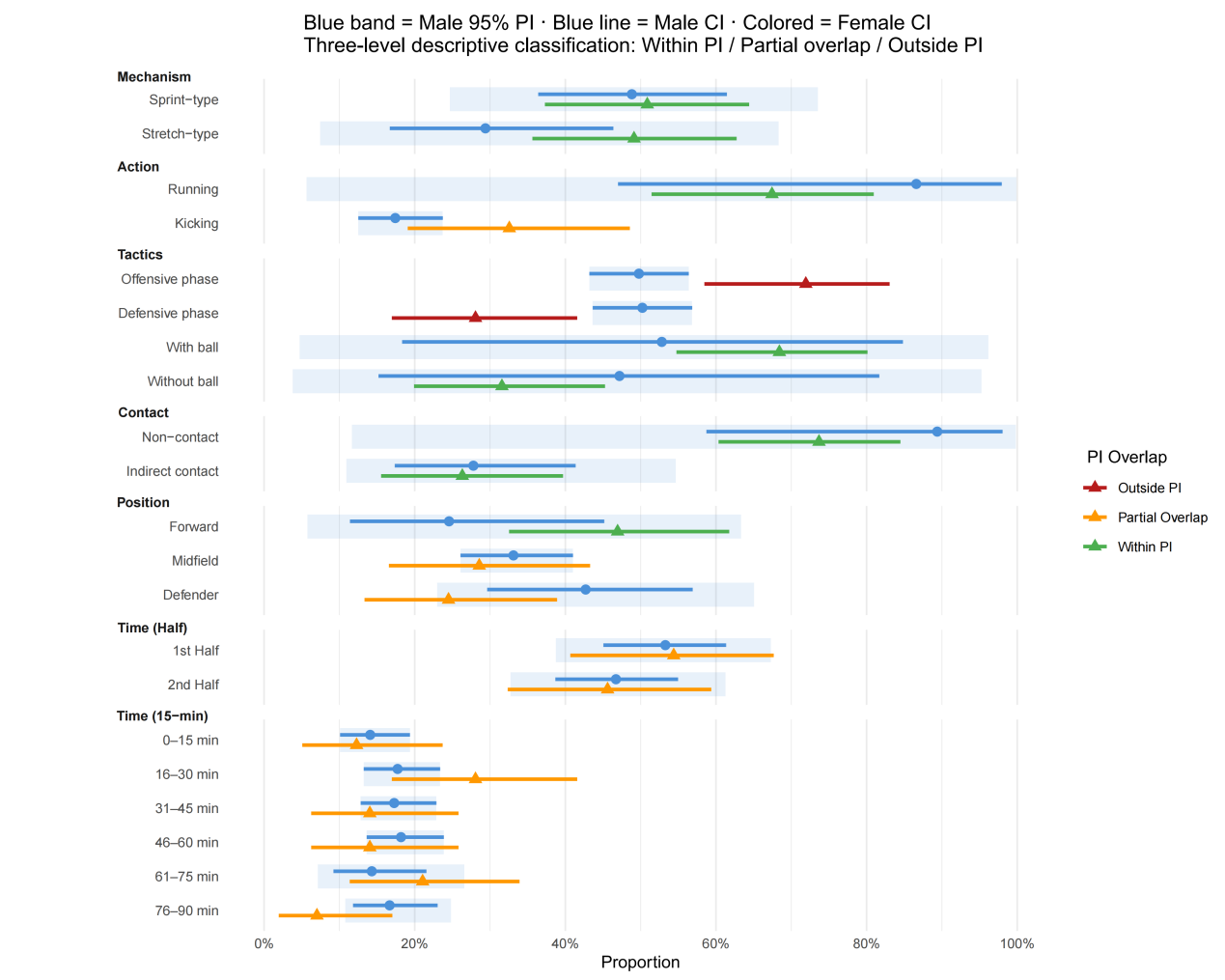


Note: This figure summarises the descriptive location of the female single-study proportion estimates and their 95% confidence intervals relative to the corresponding male 95% prediction intervals across the prespecified classification domains. The categories “within PI,” “partial overlap,” and “outside PI” are used only to describe the positional relationship between estimates. This figure does not constitute formal statistical testing, does not estimate a within-study sex effect, and should not be interpreted as confirmatory evidence of sex-specific differences.

**Supplementary Figure S6. Comparison of unadjusted and multiplicity-adjusted P values**


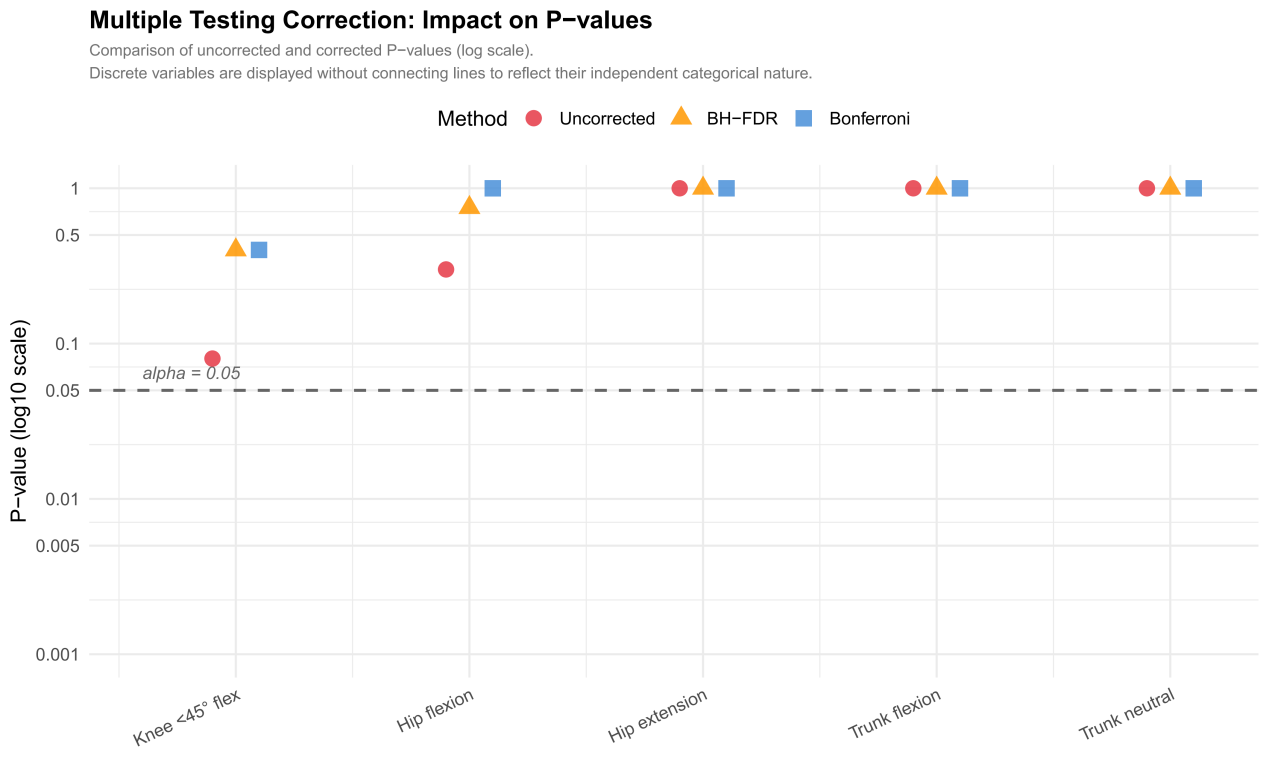


Note: This figure compares P values for discrete kinematic variables under three approaches: unadjusted, Benjamini–Hochberg false discovery rate adjustment, and Bonferroni correction. The horizontal dashed line indicates the nominal significance threshold (α = 0.05). Points represent variable-specific P values, and the y axis is displayed on a log10 scale.

**Supplementary Figure S7. Sensitivity analysis for knee-angle reclassification scenarios in the exploratory cross-study contrast**


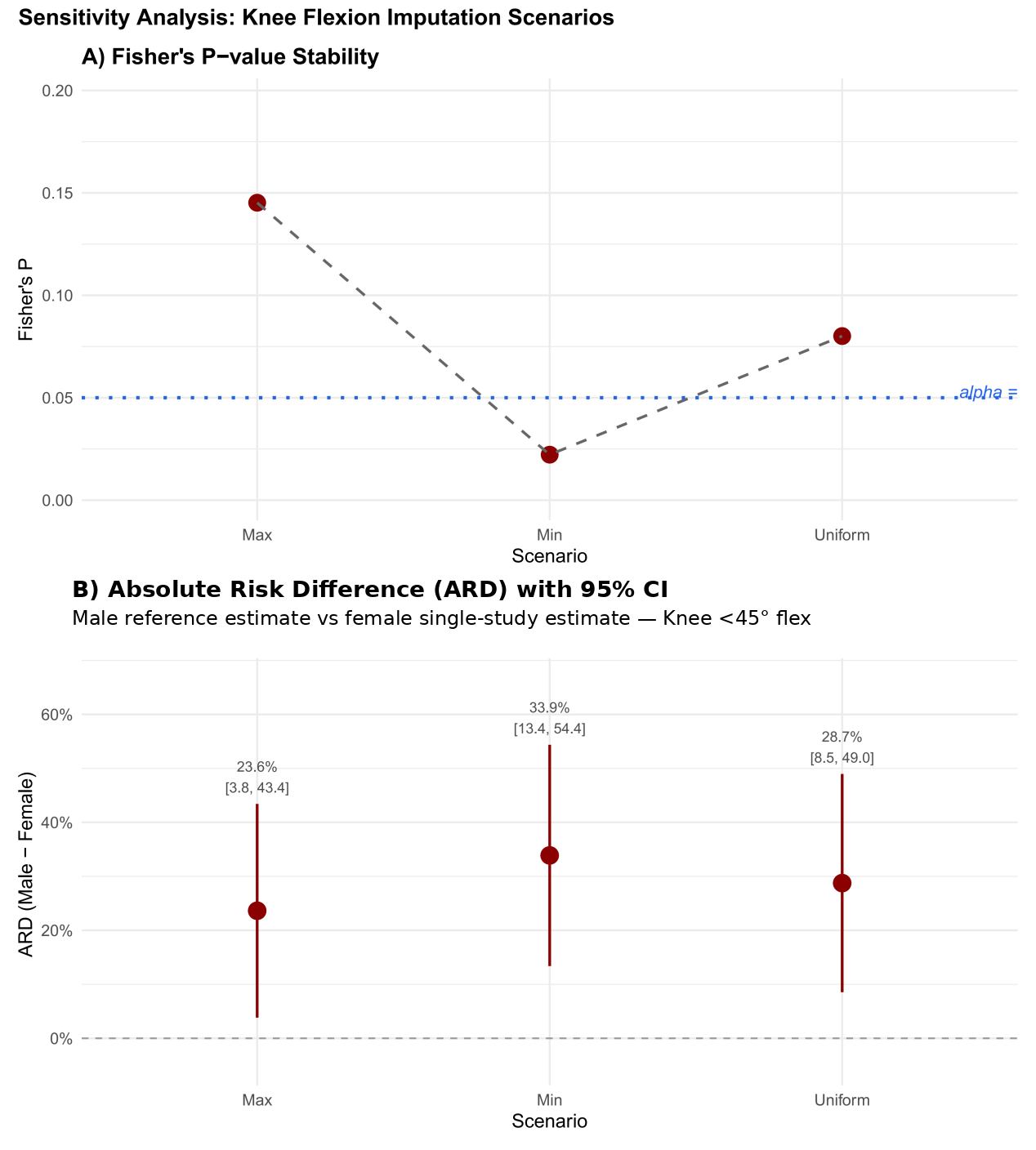


Note: This two-panel figure shows the influence of alternative reclassification scenarios for missing or inconsistently binned “knee flexion <45°” data on the exploratory cross-study contrast between the male reference estimate and the female single-study estimate. Panel A presents Fisher exact-test P values under the Max, Min, and Uniform scenarios, with the horizontal dashed line indicating the nominal significance threshold (α = 0.05). Panel B presents the corresponding absolute risk differences (ARD, male reference estimate minus female single-study estimate) and 95% CIs. These results are intended only as sensitivity analyses for the exploratory kinematic comparison and should not be interpreted as confirmatory sex-effect estimates or as formal mechanism-stratified comparisons.

**Supplementary Figure S8. Leave-one-out heterogeneity diagnostics for additional pairwise comparisons**


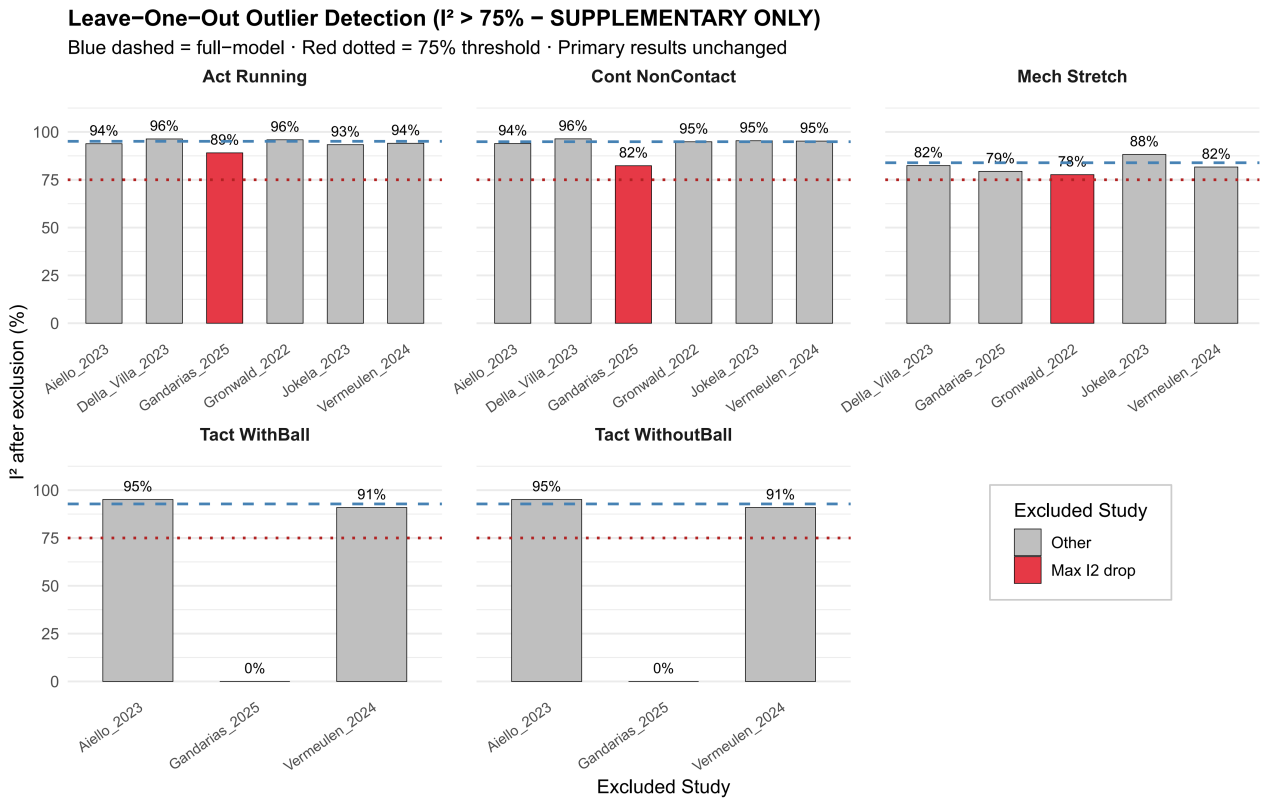


Note: This leave-one-out sensitivity figure shows changes in I² after sequential exclusion of individual studies in additional pairwise comparisons with high between-study heterogeneity. Each bar represents the recalculated I² after removal of one study. The blue dashed line indicates the heterogeneity level of the full model, and the red dotted line indicates the prespecified threshold for high heterogeneity (I² = 75%). Red bars indicate the exclusion scenario associated with the greatest reduction in I² and are shown to facilitate identification of potential outlying studies.

**Supplementary Figure S9. Funnel plots for pairwise OR meta-analyses**


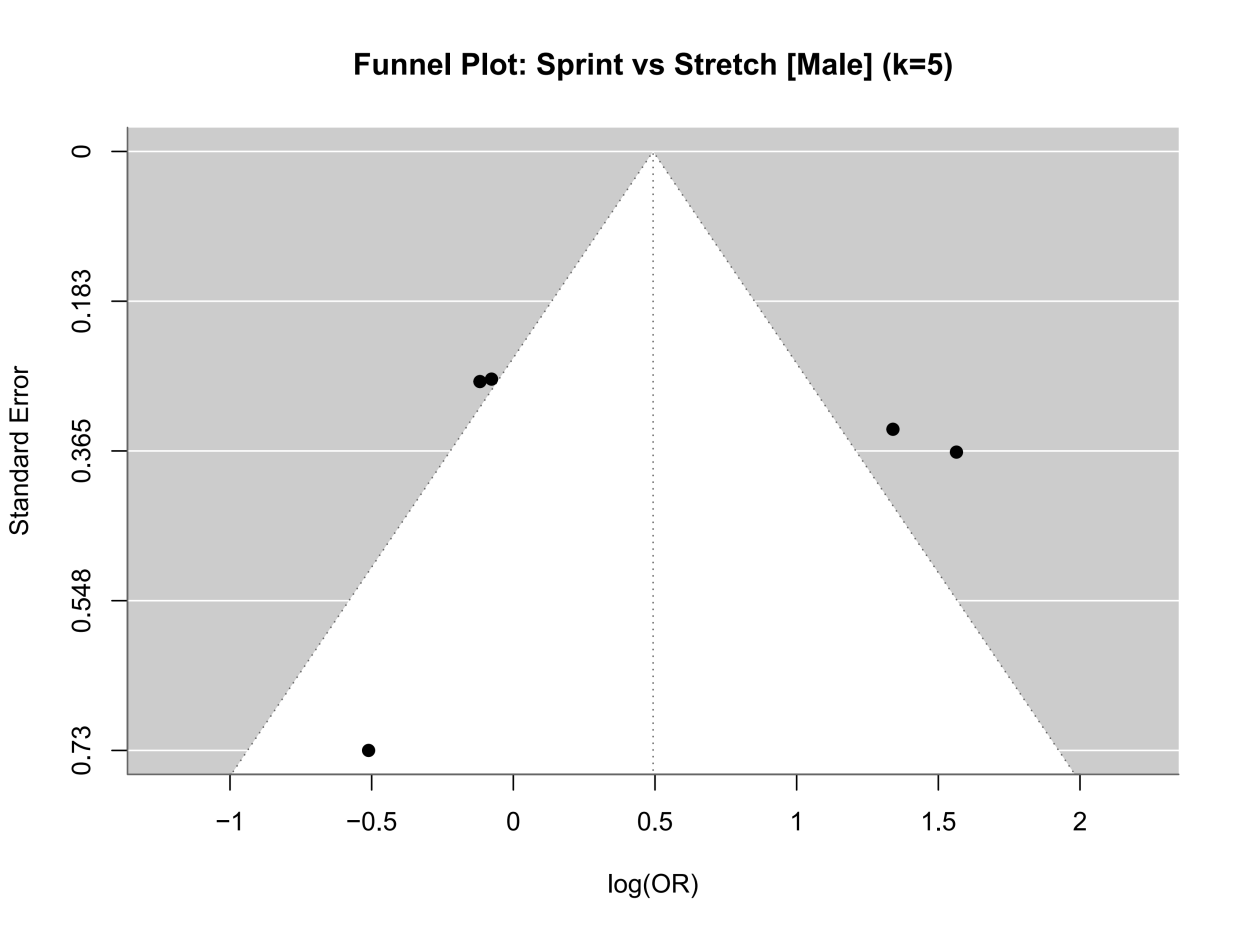


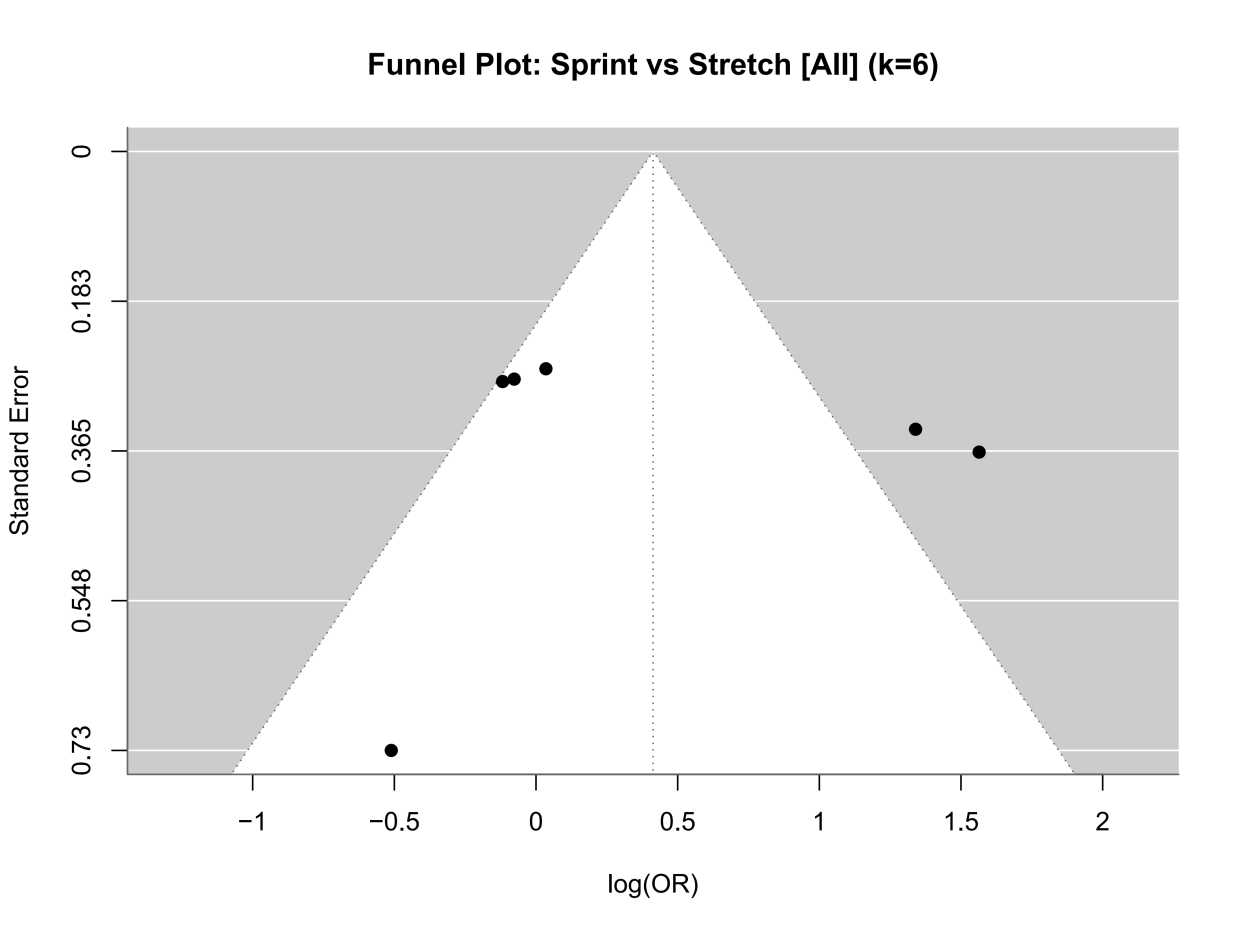


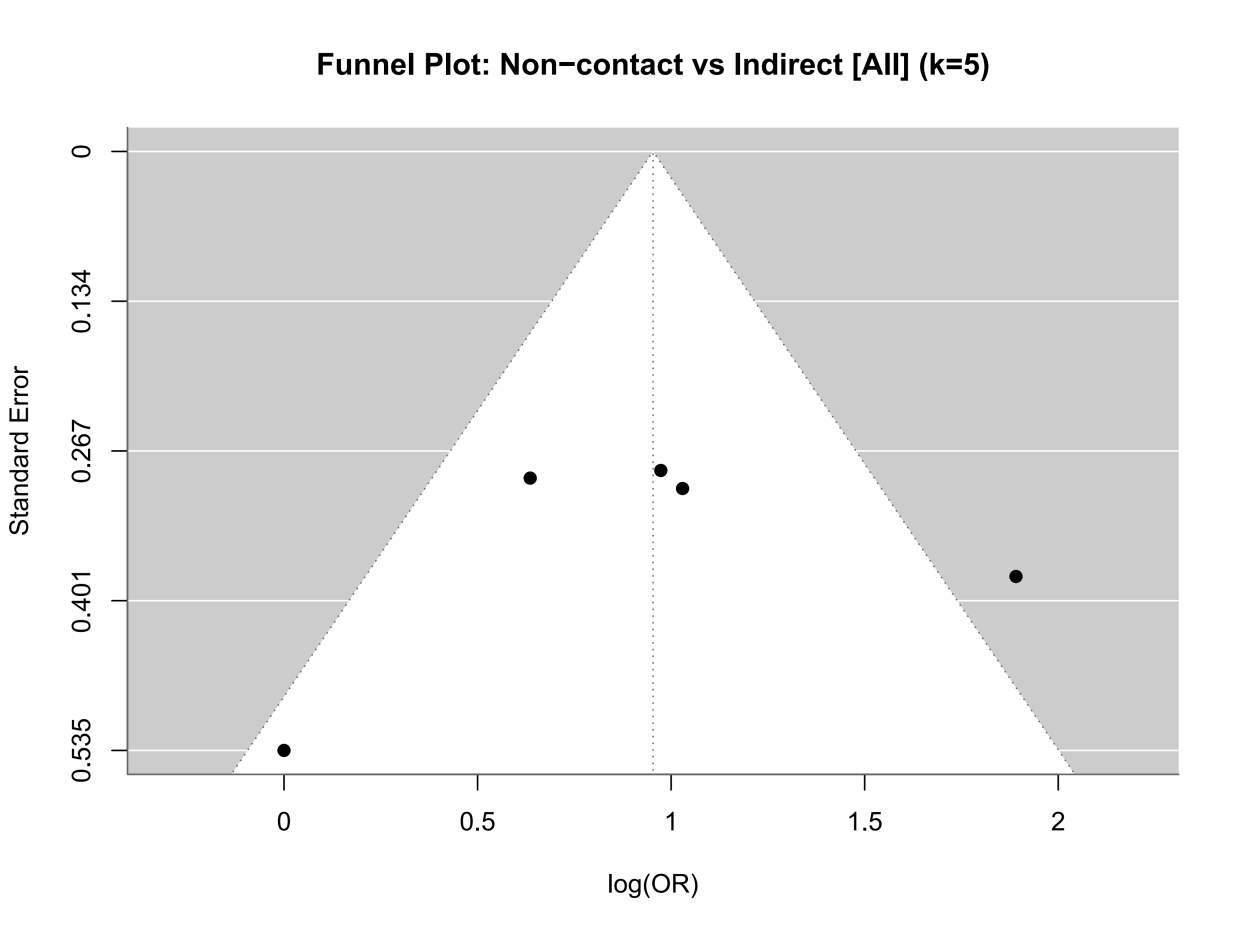


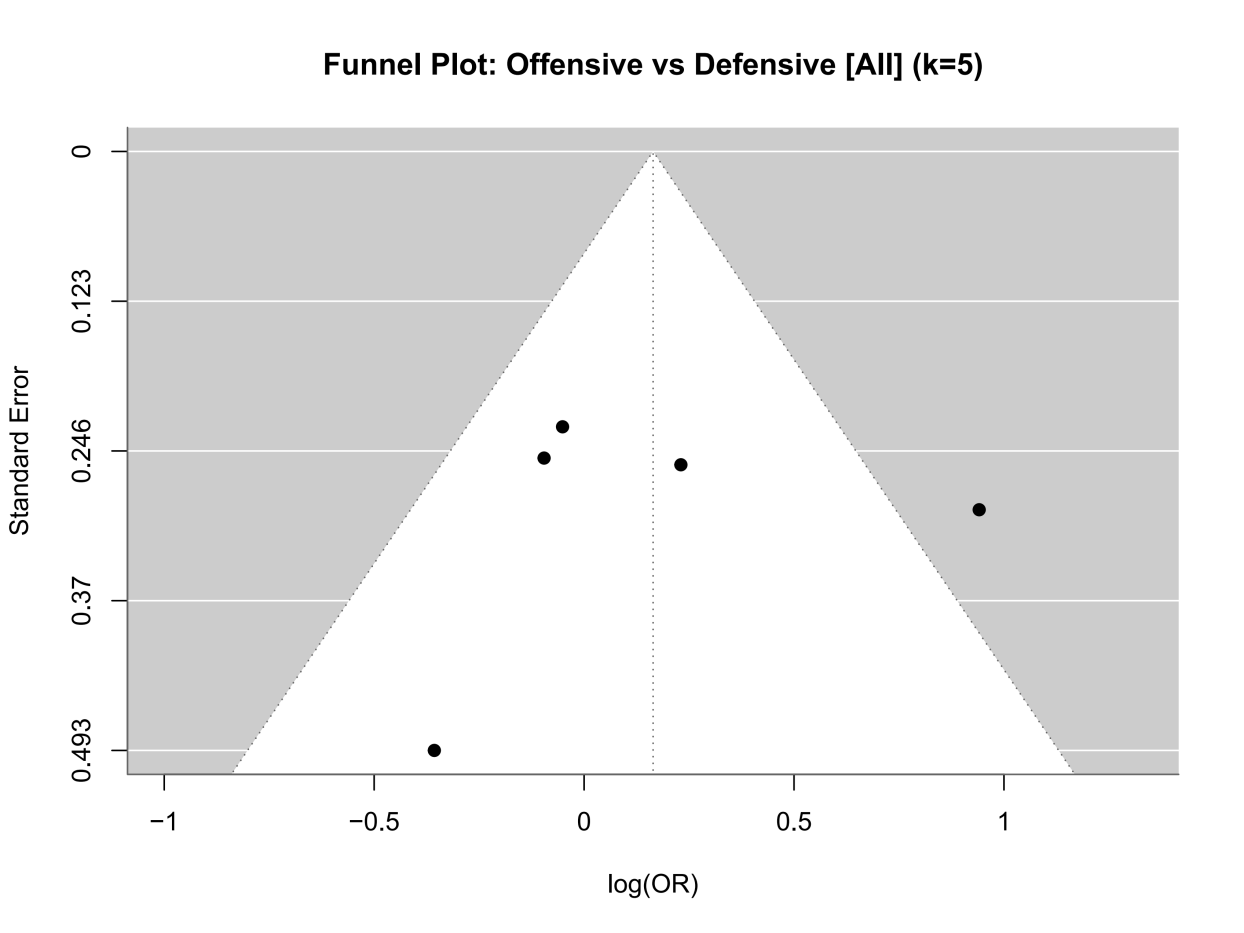


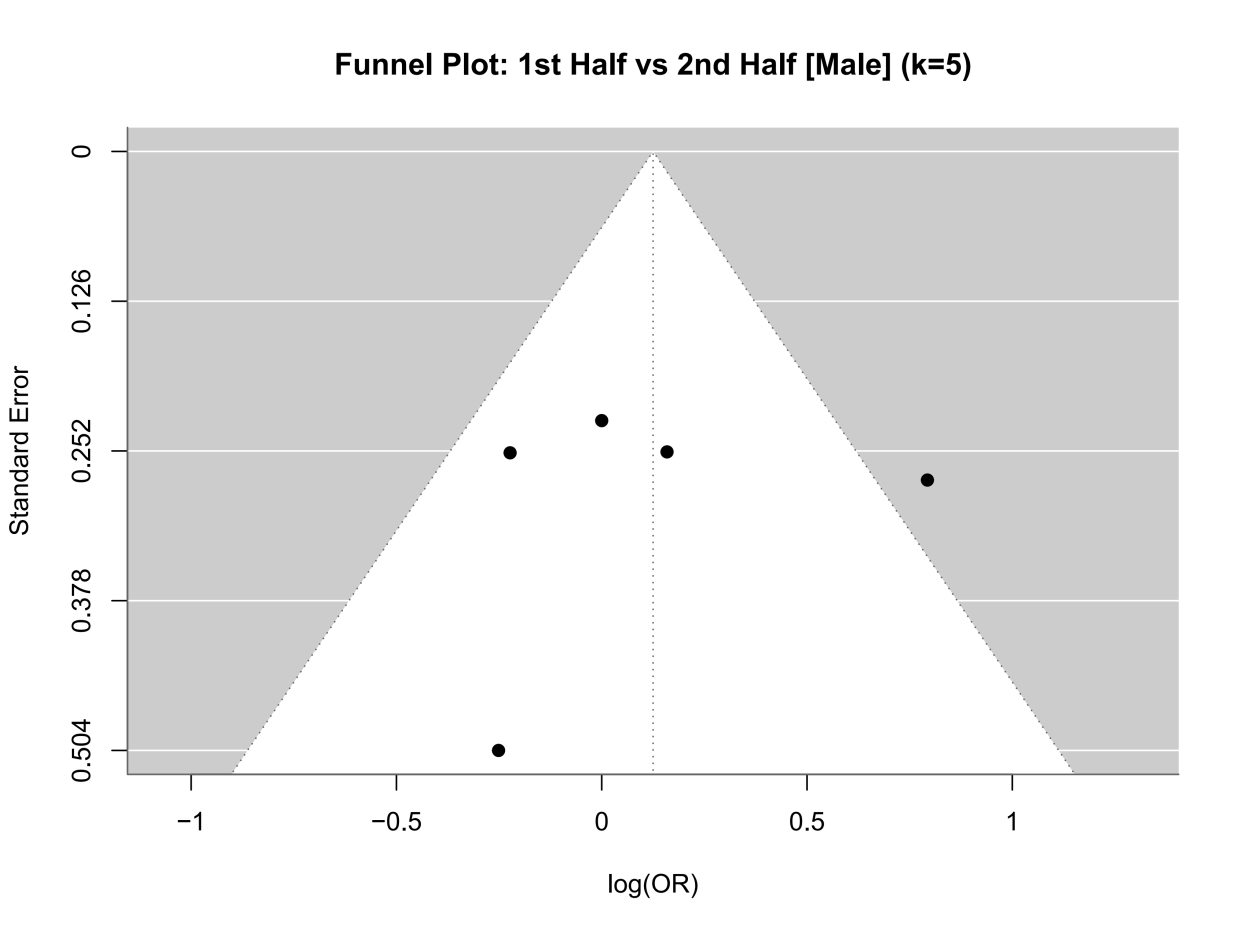


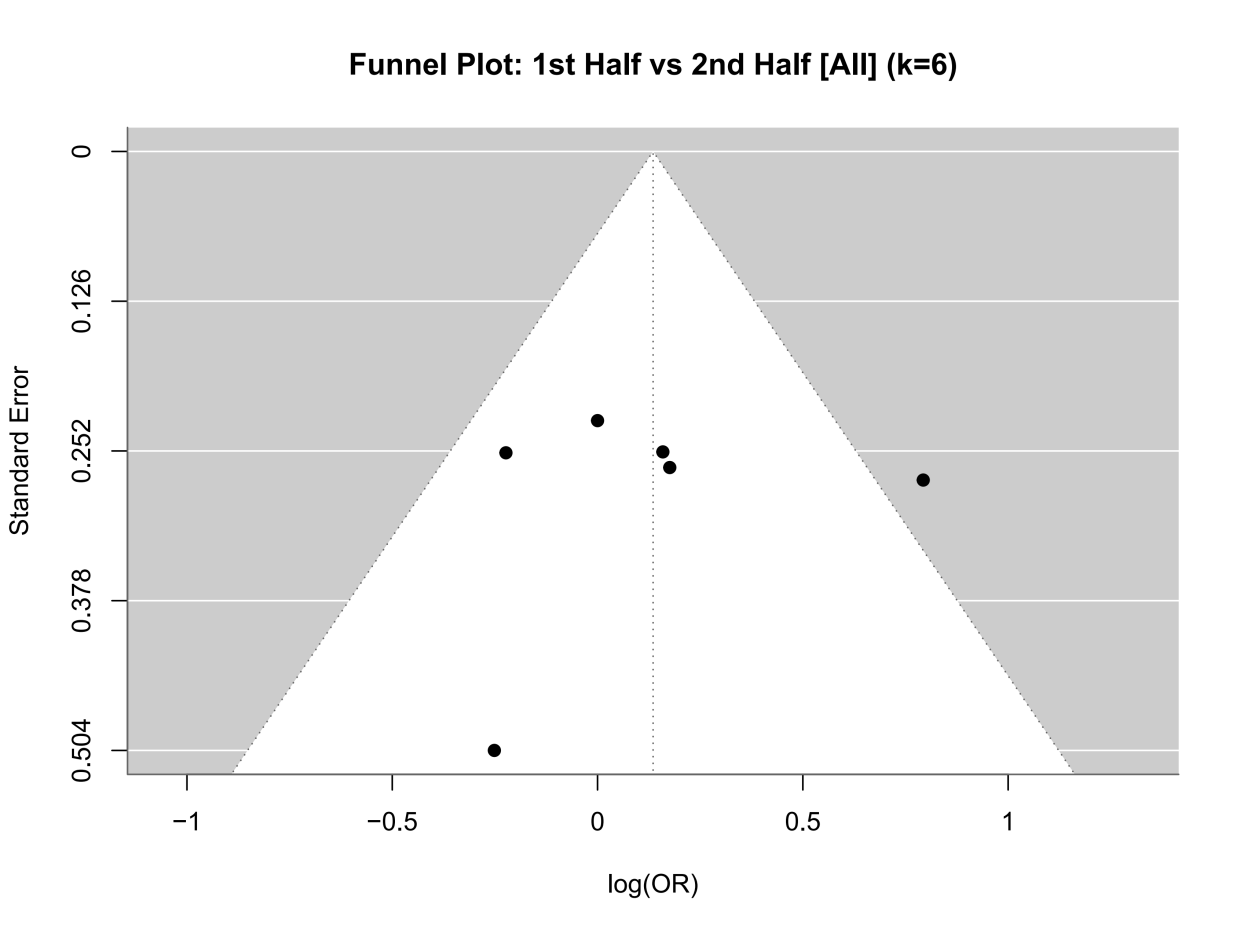


Note: Each point represents one study. The x axis shows the effect size as log(OR), and the y axis shows the standard error. The central vertical dashed line indicates the pooled effect estimate, and the diagonal boundaries indicate pseudo-95% confidence limits. These funnel plots are provided only as supplementary visual displays. Because fewer than 10 studies were included in each comparison, visual inspection and formal asymmetry testing were considered underpowered and potentially misleading; therefore, no formal inference regarding small-study effects or publication bias was made.

**Supplementary Text S1 Search strategy**

Note: Search strategies were adapted to the syntax of each database. Minor variations in term inclusion (e.g., "Kinematics" was added in PubMed, CINAHL, and Cochrane Library to maximize retrieval in those platforms) reflect interface-specific adaptations rather than conceptual differences. All searches were conducted on 12 February 2026 without language or date filters.

## PubMed

#1："Hamstring"[Title/Abstract] OR "Hamstring strain"[Title/Abstract] OR "Hamstring injury"[Title/Abstract] OR "Hamstring injuries"[Title/Abstract] OR "Hamstring muscle"[Title/Abstract]

#2："Video analysis"[Title/Abstract] OR "Video-based"[Title/Abstract] OR "Video based"[Title/Abstract] OR "Biomechanics"[Title/Abstract] OR "Kinematics"[Title/Abstract]

#3："Football"[Title/Abstract] OR "Soccer"[Title/Abstract]

#4：#1 AND #2 AND #3

## **SPORTDiscus (EBSCOhost)**

#1："Hamstring" OR "Hamstring strain" OR "Hamstring injury" OR "Hamstring injuries"

#2："Video analysis" OR "Video-based" OR "Video based" OR "Biomechanics"

#3："Football" OR "Soccer"

#4：#1 AND #2 AND #3

## **Web of Science (WoS)**

#1：TS=("Hamstring" OR "Hamstring strain" OR "Hamstring injury" OR "Hamstring injuries")

#2：TS=("Video analysis" OR "Video-based" OR "Video based" OR "Biomechanics")

#3：TS=("Football" OR "Soccer")

#4：#1 AND #2 AND #3

## **Scopus**

#1：TITLE-ABS-KEY("Hamstring" OR "Hamstring strain" OR "Hamstring injury" OR "Hamstring injuries")

#2：TITLE-ABS-KEY("Video analysis" OR "Video-based" OR "Video based" OR "Biomechanics")

#3：TITLE-ABS-KEY("Football" OR "Soccer")

#4：#1 AND #2 AND #3

## **Embase (Elsevier)**

#1：'hamstring' OR 'hamstring strain' OR 'hamstring injury' OR 'hamstring injuries'

#2：'video analysis' OR 'video-based' OR 'video based' OR 'biomechanics'

#3：'football' OR 'soccer'

#4：#1 AND #2 AND #3

## **CINAHL**

#1："Hamstring" OR "Hamstring strain" OR "Hamstring injury" OR "Hamstring injuries"

#2："Video analysis" OR "Video-based" OR "Video based" OR "Biomechanics" OR "Kinematics"

#3："Football" OR "Soccer"

#4：#1 AND #2 AND #3

## **Cochrane Library**

#1：hamstring* OR "hamstring strain" OR "hamstring injury"

#2："video analysis" OR "video-based" OR biomechanics OR kinematics

#3：football OR soccer

#4：#1 AND #2 AND #3
